# OxLDL drives pro-inflammatory and foam cell-prone macrophage reprogramming via NF-κB/HIF-1β-dependent metabolic rewiring

**DOI:** 10.3389/fcvm.2026.1957621

**Published:** 2026-10-07

**Authors:** Maozhuo Lan, Yangyang Zhou, Yue Sun, Xingcheng Yi, Mengtuan Long, Dirk M. Hermann, Fengyan Jin, Yun Dai

**Affiliations:** 1 Laboratory of Cancer Precision Medicine, Institute of Translational Research, The First Hospital of Jilin University, Changchun, Jilin, China; 2 Jilin Provincial Key Laboratory of Precision Medicine for Age-Related Diseases, Changchun, China; 3 Department of Neurology, The First Hospital of Jilin University, Changchun, Jilin, China; 4 Department of Neurology, University Hospital Essen, University of Duisburg-Essen, Essen, Germany; 5 Department of Hematology, The First Hospital of Jilin University, Changchun, Jilin, China; 6 Department of Hematology and Oncology, Shanghai Jing'an District Zhabei Central Hospital, Shanghai, China

**Keywords:** atherosclerosis, macrophage, metabolic rewiring, oxidized LDL, transcriptional reprogramming

## Abstract

**Introduction:**

Dysregulated lipid metabolism and inflammation are two core pathological drivers of atherosclerosis; however, their causal interplay remains incompletely elucidated. Macrophages play a central role in sustaining unresolved inflammation, which is a hallmark of atherosclerosis. Recent single-cell RNA-sequencing studies have revealed phenotypic heterogeneity among macrophages in atherosclerotic lesions, encompassing two key pro-atherogenic subsets: pro-inflammatory and foam cell-forming macrophages. Given that oxidized low-density lipoprotein (oxLDL) is a pivotal pathological factor in atherosclerosis, we investigated whether and how oxLDL drives the reprogramming of macrophages toward a pro-atherogenic phenotype.

**Methods:**

Transcriptomic analyses were performed on macrophages exposed to oxLDL, followed by a series of *in vitro* and *ex vivo* experiments to characterize the phenotypic, metabolic, and functional changes.

**Results:**

We identified a macrophage phenotype, named oxLDL-activated macrophages (OAMs), which displayed significant molecular and functional similarities to both pro-inflammatory and foam cell-forming macrophages. OxLDL-induced macrophage reprogramming was associated with a metabolic paradigm shift from oxidative phosphorylation to glycolysis, which was abrogated by inhibition of NF-κB activation, accompanied by polarization toward an anti-inflammatory phenotype. Knockdown of *ARNT* (encoding HIF-1β) recapitulated the effects of NF-κB inhibition, positioning HIF-1β downstream of the NF-κB pathway. Furthermore, inhibition of NF-κB also attenuated oxLDL-induced inflammasome activation and priming for a heightened secondary response, impaired the foam cell-forming capacity of OAMs, and moderately affected efferocytosis.

**Conclusions:**

Our findings demonstrate that oxLDL drives macrophage polarization toward a pro-atherogenic (pro-inflammatory and foam cell-prone) phenotype via metabolic rewiring and transcriptional remodeling mediated by HIF-1β-dependent crosstalk between the NF-κB and HIF pathways. This signaling axis may serve as a potential therapeutic target for atherosclerosis.

## 1 Introduction

Atherosclerosis is the core pathological basis of lethal cardiovascular and cerebrovascular diseases, including coronary heart disease (CHD), ischemic stroke (IS), and peripheral arterial disease, ranking first among global chronic disease-induced mortality causes (1). Epidemiological data have revealed that dyslipidemia-mediated atherosclerotic cardiovascular diseases cause over 18 million annual deaths worldwide, and morbidity is on the rise among young and middle-aged populations, resulting in a severe socioeconomic burden (1). Clinically, statin-based lipid-lowering treatment can partially delay plaque progression; however, nearly 30% patients experience residual cardiovascular events despite standardized lipid control, indicating that single lipid regulation cannot fundamentally block atherogenic progression. Pathologically, AS is defined as a chronic arterial inflammatory disorder triggered by lipid metabolic disorders, characterized by vascular lipid deposition, immune cell infiltration, and irreversible vascular remodeling. However, the molecular link between lipid dysfunction and sustained vascular inflammation remains unclear (2). Lesional macrophage polarization dominates the progression of atherogenesis: pro-inflammatory M1 macrophages aggravate vascular endothelial injury and plaque inflammation, while anti-inflammatory M2 macrophages facilitate inflammation resolution and stabilize vulnerable plaques (3). Recent human plaque single-cell RNA sequencing (scRNA-seq) has further revised the traditional M1/M2 binary classification, identifying two plaque-specific pathogenic macrophage subsets, pro-inflammatory macrophages and TREM2^hi^ foamy macrophages, which jointly determine plaque rupture risk (4). However, the upstream lipid stimulus and core signaling cascade that induce such pathological macrophage polarization remain unclear, becoming the key bottleneck restricting targeted therapy for atherosclerotic diseases (5).

Redox signaling is a well-documented regulator of monocyte and macrophage biology, involving survival, differentiation, migration, and function (6). Moreover, reactive oxygen species (ROS) play an important role in metabolic/inflammatory diseases, including atherosclerosis (7). Under excessive oxidative stress in a plaque microenvironment, native low-density lipoprotein (nLDL) undergoes lipid peroxidation to form cytotoxic oxidized low-density lipoprotein (oxLDL), which bridges dyslipidemia and arterial inflammation in the necrotic core and lipid-rich area of human atherosclerotic plaques (8–10). Classically, macrophages engulf extracellular oxLDL through surface scavenger receptors, such as CD36 and LOX-1, and accumulate intracellular lipid droplets to form foamy macrophages, accelerating plaque lipid deposition and necrotic core formation (11). Apart from lipid metabolism disturbance, accumulating evidence indicates that oxLDL acts as a damage-associated molecular pattern (DAMP), triggering persistent macrophage activation and pyroptosis, leading to irreversible vascular damage even after blood lipid levels return to normal (12, 13). Both acute inflammatory bursts and long-term immune memory rely on intracellular transcriptional and immunometabolic reprogramming; however, the upstream signal transduction axis coordinating these two biological processes has not been verified (14). Existing studies have separately explored oxLDL-induced inflammation and foam cell formation but have ignored the intrinsic linkage between the macrophage metabolic shift and pathogenic polarization, resulting in fragmented mechanistic conclusions.

Given the above clinical dilemmas and research gaps, this study aimed to resolve two core scientific questions: whether oxLDL drives the formation of plaque-specific pathogenic macrophage subsets and what core signaling cascade links oxLDL-induced metabolic disorder and vascular inflammation. Combined with clinical specimen verification, public omics reanalysis, and *in vitro* and *ex vivo* macrophage functional validation, this work identified an oxLDL-activated macrophage (OAM) phenotype merging molecular signatures of plaque pro-inflammatory and TREM2^hi^ foamy macrophages. Mechanistically, we further clarified that HIF-1β mediates NF-κB-HIF signaling crosstalk under normoxia, governing OAM transcriptional reprogramming and the glycolysis-dominated metabolic switch. This study complements the lipid-inflammation regulatory network of atherosclerosis, provides a novel therapeutic target for atherosclerotic vascular diseases, and offers a mechanistic explanation for residual clinical cardiovascular inflammation after lipid-lowering treatment.

## 2 Materials and methods

The full experimental procedures are provided in the Supplementary Material.

### 2.1 Cells and reagents

Murine RAW264.7 and human THP-1 monocyte cell lines were obtained from the ATCC. THP-1 cells were differentiated into macrophages through 48 h of 50 ng/mL phorbol-12-myristate-13-acetate (PMA) stimulation followed by 24 h of resting culture. Human nLDL and oxLDL were commercially acquired, prepared using standardized purification and oxidation procedures, and stored at 2–8 °C. Most cellular experiments adopted 50 μg/mL oxLDL, consistent with public transcriptome datasets. The IKK inhibitor parthenolide, IKK2 inhibitor IV, lipopolysaccharide (LPS), and nigericin were used for pathway assays.

### 2.2 Patient samples

This study was approved by the Ethics Committee of The First Hospital of Jilin University (#2019-055). The specimens included peripheral blood from 27 patients with CHD/IS and embolus samples from 4 patients with IS embolectomy (Supplementary Table S1). All enrolled patients received statins, antiplatelet agents, and/or other therapies at the time of sampling. Peripheral blood mononuclear cells were induced into macrophages using macrophage colony-stimulating factor (M-CSF). Serum cytokines and plasma oxLDL levels were detected using cytometric bead array (CBA) and ELISA, respectively. Hyperlipidemia was diagnosed based on the clinical blood lipid cutoff values.

### 2.3 Molecular biology experiments

For HIF-1β knockdown, two independent *Arnt*-targeting shRNAs (shARNT-1: 5′-CCAACAACTTCTAAGAGAT-3′; shARNT-2: 5′-CCAGACAAGCTAACCATCTTACTC-3′) and a negative control shRNA were packaged into lentiviruses, transfected into RAW264.7 cells, and selected with puromycin. Transduction efficiency was verified using green fluorescence protein (GFP).

Total cellular RNA was extracted for qPCR, with inflammatory, metabolic, and phenotypic genes detected using specific primers. GAPDH served as an internal reference, and gene relative expression was calculated using the 2^ΔΔCT^ method. Western blotting was performed to detect signaling pathway, inflammation, and pyroptosis-related protein levels, and β-actin/GAPDH was used as the loading control. Immunoprecipitation was performed to verify NLRP3 inflammasome assembly.

### 2.4 Functional detection assays

An Agilent Seahorse analyzer was used to measure the cellular oxygen consumption rate (OCR) and extracellular acidification rate (ECAR) to assess oxidative phosphorylation (OXPHOS) and glycolysis activity. Macrophage lipid uptake was observed via Oil Red O staining. Efferocytosis capacity was assessed by fluorescence staining and flow cytometry using a co-culture system. The heightened sensitivity of OxLDL-primed macrophages to secondary stimuli was verified by LPS stimulation combined with cytokine qPCR detection. Embolic tissue macrophage distribution was detected by H&E and immunohistochemistry (IHC) staining, and quantified by ImageJ software.

### 2.5 Transcriptomic and statistical analyses

All bioinformatics analyses, including K-means clustering, KEGG, and GO enrichment, were performed using the R2 Genomics Analysis Platform. The public GEO dataset SuperSeries GSE54975, encompassing the expression subseries (GSE54666) and the H3K27ac ChIP-seq subseries (GSE54972), was adopted for human macrophage transcriptome and ChIP-seq reanalysis to identify and characterize OAMs (15). An independent murine macrophage transcriptome dataset (GSE139439) was used to validate OAMs (11). All *in vitro* experiments were performed at least three times. Data are presented as mean ± SD. Two-tailed *t*-test and one-way ANOVA were used for significance analyses. *P* < 0.05 indicated a statistical difference.

## 3 Results

### 3.1 Inflammation correlates with hyperlipidemia in atherosclerosis patients

IHC staining of the embolus from patients with IS revealed a higher prevalence of CD68^+^ macrophages compared to CD163^+^ M2 macrophages (Figure 1a; Supplementary Figure S1). Quantitative analysis demonstrated that the average optical density (AOD) of CD68 was significantly higher than that of CD163 across four independent patient embolus specimens (*P* < 0.01; Figure 1b; Supplementary Figure S1), reflecting increased recruitment or reduced egress, alongside impaired resolution of inflammation. For clinical serological detection, hyperlipidemic patients who had been receiving statins, antiplatelet agents, and/or other therapies at sampling (Supplementary Table S1) exhibited elevated plasma oxLDL concentrations relative to normolipidemic healthy donors, although the difference was not statistically significant (*P* = 0.078; Figure 1c). Serum CBA assays further validated that circulating pro-inflammatory cytokines, including TNF-α, IL-1β, IL-6, and IL-8, were upregulated in hyperlipidemic patients, whereas the level of anti-inflammatory IL-10 remained comparable between the two cohorts (Figure 1d). Pearson correlation analysis confirmed that serum LDL-c levels were positively correlated with TNF-α (*r* = 0.520, *P* = 0.016), IL-1β (*r* = 0.488, *P* = 0.025), IL-6 (*r* = 0.481, *P* = 0.027), and IL-8 (*r* = 0.426, *P* = 0.054), but not with IL-10 (*r* = 0.141, *P* = 0.54) and IL-12 (*r* = 0.315, *P* = 0.16). Collectively, dyslipidemia-related oxLDL elevation is associated with both lesional and systemic inflammation.

**Figure 1:**
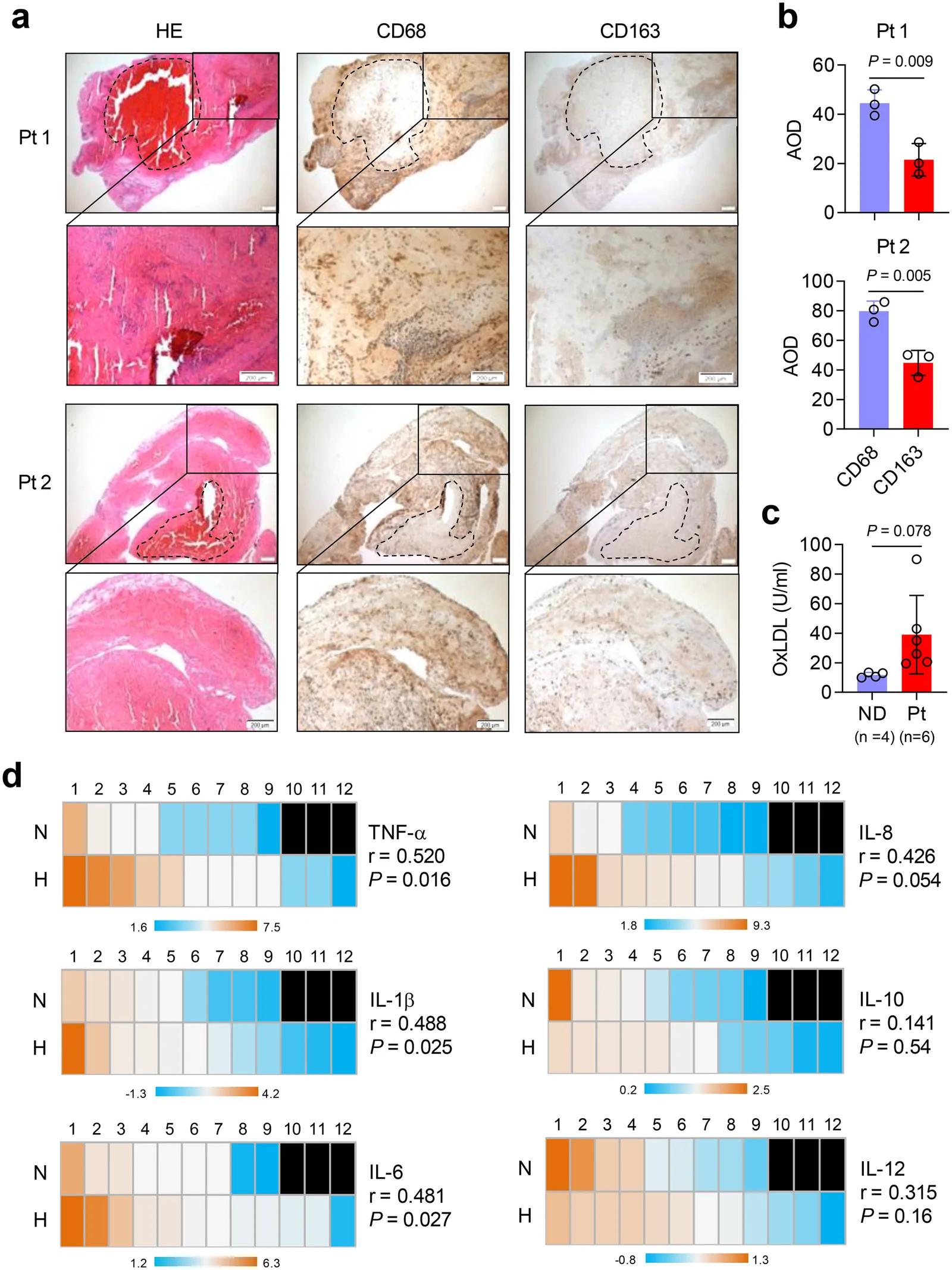
Lesional inflammation is associated with lipid abnormalities in atherosclerosis. (**a,b**) Examples of IHC staining in emboli of patients (Pt) with IS (scale bar = 200 μm; dashed line indicates blood clots). AOD, average optical density (two-tailed Student's *t*-test). **(c)** ELISA-based analysis of plasma oxLDL in the peripheral blood in patients with CHD with hyperlipidemia (Pt) and healthy donors (ND; two-tailed Student's *t*-test). **(d)** CBA of serum cytokines in patients with CHD/IS with hyperlipidemia (H; *n* = 12) or normal lipid levels (N; *n* = 9) who had been receiving statins, antiplatelet agents, and/or other therapies at the time of sampling (Supplementary Table S1). The color bar indicates cytokine concentrations (pg/mL, log2). Correlation between cytokines and LDL cholesterol using Pearson’s coefficient.

### 3.2 OxLDL induces a pathogenic macrophage phenotype (OAM)

Macrophage polarization is primarily governed by transcriptional reprogramming (16). Transcriptome analysis (GSE54666) showed that oxLDL stimulation triggered 169 upregulated and 160 downregulated differentially expressed genes (DEGs) in primary human monocyte-derived macrophages (false discovery rate/FDR < 0.05, |log2FC| > 1; Figure 2a; Supplementary Figure S2a). The volcano plot shows the sorted upregulated and downregulated DEGs (Figure 2b). KEGG enrichment showed that upregulated DEGs were predominantly enriched in the PPAR signaling pathway, while downregulated genes clustered in steroid biosynthesis (Figure 2c; Supplementary Figures S2b,c). Distinct from classical LPS-induced M1 and IL-4-induced M2 macrophages (17), OAMs exhibited notable molecular characteristics (Figure 2d; Supplementary Figures. S2d,e). They showed upregulated inflammatory receptors (*IL1R1*, *IL6R*), *NLRC4* (an inflammasome regulator) (18), and *DFNA5*/GSDME (a mediator of pyroptosis) (19), without induction of the canonical M1 marker *NOS2*/iNOS and *TLR4* (a key LPS receptor mediating NF-κB signaling) (20). They also suppressed M2 signature genes, including M2 markers (*CD163*, *CLEC7A*/DECTIN-1, *CD209*/DC-SIGN, *CD200R*, *MS4A4A*), anti-inflammatory cytokines (*IL10*), cytokine receptors (*TGFBR1*, *TGFBR2*), signaling molecules linked to M2a–d subsets (*TLR8* and *CCL23*), and *CCR7* (mediating foam cell egress during atherosclerotic plaque regression) (21). In addition, they upregulated foam cell-related receptors (*CD36*, *VLDLR*), accompanied by activated cholesterol efflux signals mediated by the LXRα-ABCA1/ABCG1 axis (22). OAMs were also different from reported atherosclerosis-related M4 and Mhem subsets, while sharing certain genes (e.g., *SRXN1* and *TXNRD1*, but not *HMOX1*) with Mox (Supplementary Figure S2f), a phenotype of macrophages in response to oxidized phospholipids (23, 24). Notably, OAMs expressed feature genes of TREM2^hi^ foamy (e.g., *CTSD*, *LGALS3*, *FABP4*, but not *TREM2*) and inflammatory macrophages (e.g., *CEBPB*, but not *NLRP3, NFKBIZ* and *CXCL2*), while downregulated those for resident-like macrophages (e.g., *F13A1*, *TXNIP*, and *SEPP1*, but not *PF4*) identified in atherosclerotic lesions using scRNA-seq (4, 5). Collectively, these data define an oxLDL-induced macrophage phenotype characterized by pro-inflammatory and foam cell-prone programs, with impaired anti-inflammatory function.

**Figure 2:**
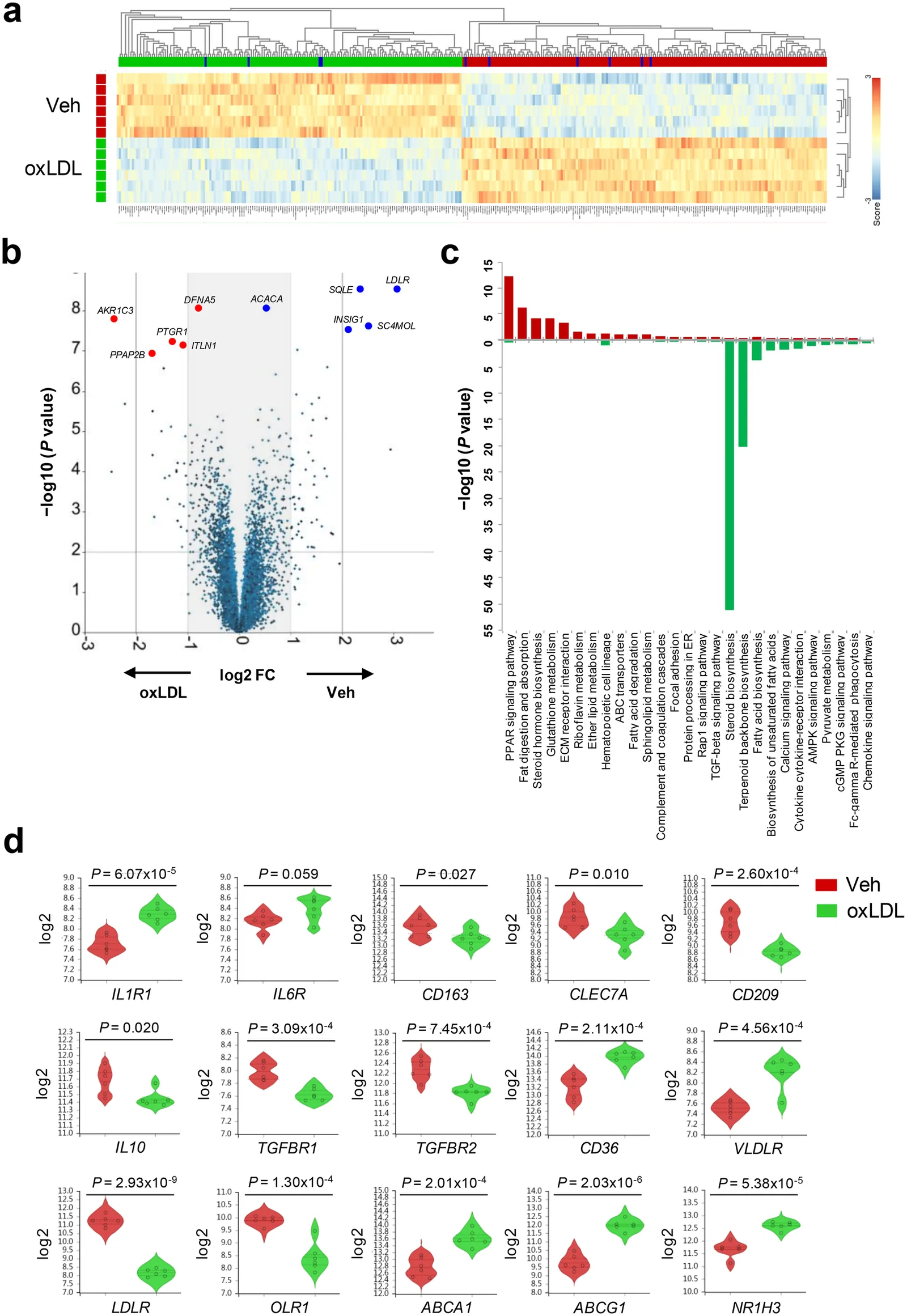
Transcriptomic analysis reveals a unique gene expression profile (GEP) of macrophages in response to oxLDL. GEP analysis of primary human monocyte-differentiated macrophages exposed to 50 μg/mL oxLDL vs. vehicle (Veh) for 48 h (*n* = 6 for each condition; GEO ID: GSE54666). **(a,b)** All DEGs (log2 FDR, cutoff, *P* < 0.05). Heatmap **(a)** and volcano graph **(b)**. FC, fold change. **(c)** KEGG analysis of DEG-enriched pathways (red, upregulation; green, downregulation; *t*-test, cutoff, *P* < 0.0001). **(d)** Expression of representative genes (log2, one-way ANOVA).

### 3.3 OxLDL triggers macrophage pro-inflammatory polarization and glycolytic metabolic switch

In murine RAW264.7 cells, 50 μg/mL oxLDL upregulated M1 markers: *Nos2* (4.7-fold), *Il1b* (9.3-fold), *Tnfa* (9.7-fold), and *Ccl2*/MCP-1 (40.6-fold), without altering M2 marker *Arg1* transcription (Figure 3a); equivalent concentration of nLDL induced similar but milder inflammatory changes, with negligible MCP-1 activation. Consistently, oxLDL treatment elevated the protein abundance of total and mature IL-1β in human THP-1-derived macrophages (Figure 3b). For patient-derived primary macrophages (purity >90%; Supplementary Figure S3a), 24 h of oxLDL incubation induced contracted cellular protrusions, increased cytoplasmic granularity under bright-field microscopy (Figure 3c; Supplementary Figure S3b), and upregulated iNOS, IL-1β, and, to a lesser extent, TNF-*α* and MCP-1, alongside suppressed expression of the M2 marker CD206 (Figure 3d). Parallel treatment with equal concentrations of nLDL yielded qualitatively similar but milder transcriptional changes (*n* = 6; Supplementary Figure S3c).

**Figure 3:**
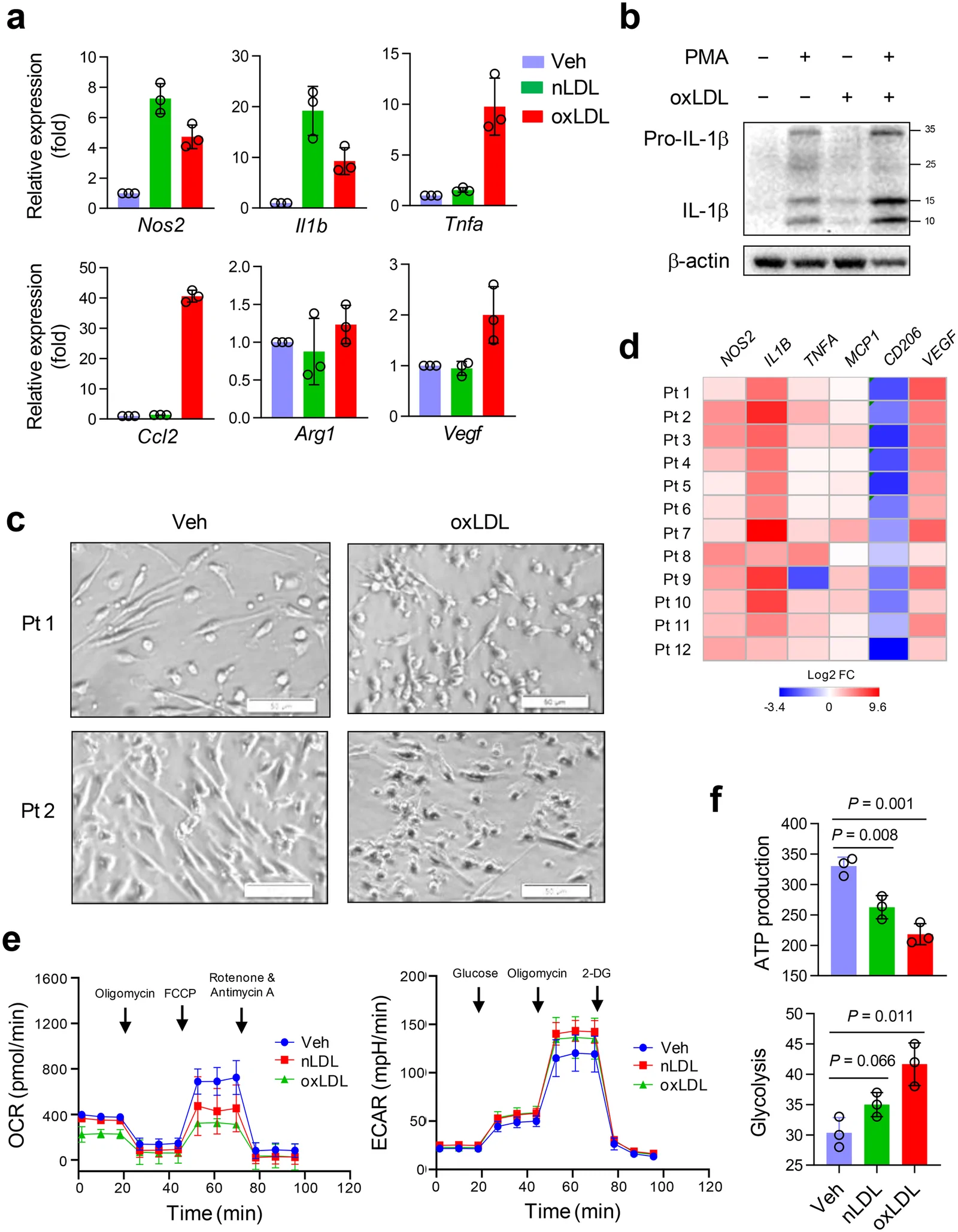
OxLDL induces pro-inflammatory polarization of macrophages, associated with a metabolic paradigm shift. **(a)** Expression of M1 and M2 genes in RAW264.7 cells incubated with 50 μg/mL nLDL or oxLDL and vehicle as a control for 24 h. Values represent the mean ± SD of at least three independent experiments. For each experiment, the readout of the vehicle treatment is arbitrarily set to 1.0. **(b)** IL-1β maturation in THP-1-derived macrophages exposed to 50 μg/mL oxLDL for 24 h. The results of a representative experiment are shown, and an additional study yielded equivalent results. (**c,d**) Macrophages differentiated from peripheral blood monocytes of patients with CHD/IS (*n* = 12) after exposure to 10 μg/mL oxLDL vs. vehicle for 24 h (scale ba*r* = 50 μm). Representative microscopic images **(c)** and heatmap for M1 and M2 gene expression [**(d)** qPCR, fold change for oxLDL vs. vehicle]. (**e,f**) Example of Seahorse analysis for OCR and ECAR in THP-1-derived macrophages incubated with 50 μg/mL nLDL or oxLDL vs. vehicle for 24 h **(e)**. Average values of mitochondrial ATP production and glycolysis. Values represent the mean ± SD of at least three independent experiments. Two-tailed Student's *t*-test **(f)**.

Metabolically, Seahorse assays revealed that oxLDL inhibited macrophage basal OCR, maximal respiratory capacity, and mitochondrial adenosine triphosphate (ATP) production, whereas it elevated the basal ECAR and glycolytic reserve (Figures 3e,f), shifting cellular energy metabolism from OXPHOS to anaerobic glycolysis (25). nLDL treatment triggered an identical metabolic tendency but only half the amplitude of oxLDL-induced metabolic alterations (Figures 3e,f). KEGG pathway enrichment of oxLDL-responsive DEGs highlighted the robust remodeling of central energy metabolism, encompassing OXPHOS, tricarboxylic acid (TCA) cycle, and glycolysis/gluconeogenesis modules (Supplementary Figure S3d). Transcriptome verification confirmed that core OXPHOS subunits (*NDUFA8* and *ATP6V1E1*) and TCA cycle rate-limiting enzymes (*IDH1* and *IDH2*) were downregulated, while glycolysis initiation genes *HK1* and *HK2* and downstream metabolic enzymes *ALDH3A2* were activated (Supplementary Figure S3e). These data demonstrate that oxLDL skews macrophages toward a pro-inflammatory state with diminished anti-inflammatory capacity, coupled with the metabolic rewiring characteristic of M1 polarization.

### 3.4 OxLDL synchronously activates NF-κB and HIF signaling pathways

OxLDL-activated NF-κB and HIF pathway core genes at the transcriptional level (GSE54666; Figures 4a,b), and ChIP-seq reanalysis (GSE54972) validated increased H3K27ac epigenetic modification peaks at enhancer regions of *HIF1A*, *ARNT*, *RELA*, *EPAS1*, and *CHUK* loci (Supplementary Figure S4a), indicating enhanced chromatin accessibility and transcriptional activation of dual pathways. In RAW264.7 cells, 25–100 μg/mL oxLDL (2 h) triggered dose-dependent IKKα/β and p65 phosphorylation, and the time-course assay proved that NF-κB activation occurred as early as 30 min (Figure 4c). Both canonical NF-κB upstream mediator IKKα/β phosphorylation and non-canonical pathway mediators TRAF3 and NIK were synchronously upregulated (Figure 4c; Supplementary Figure S4b). Meanwhile, oxLDL induced a time-dependent accumulation of HIF-1α and HIF-1β proteins under normoxia (Figure 4d; Supplementary Figure S4c) (26). Comparable but milder pathway activation was observed in RAW264.7 cells treated with equivalent concentrations of nLDL (Supplementary Figure S4d). Longer exposure to oxLDL (24 h) sustained dual pathway activation, although the robustness of activation was reduced relative to the early time points described above in both murine and human macrophages (Figure 4e; Supplementary Figure S4e). In summary, oxLDL induces rapid, dose-dependent, and persistent co-activation of the NF-κB and HIF pathways, independent of hypoxia.

**Figure 4:**
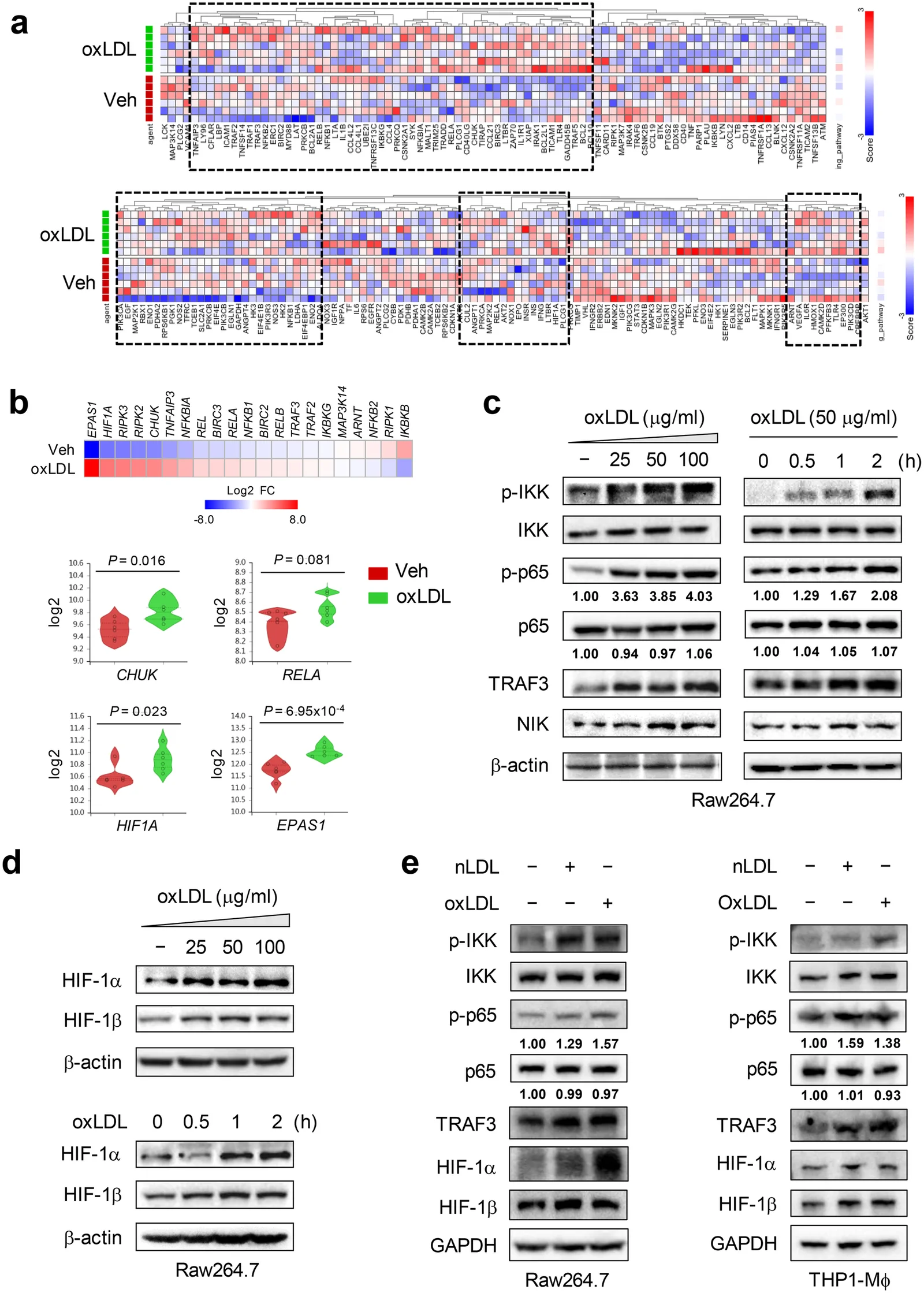
OxLDL activates both NF-κB and HIF pathways in macrophages. (**a,b**) DEGs (log2) enriched for the NF-κB (upper) or HIF pathways (lower) in primary human monocyte-differentiated macrophages exposed to 50 μg/mL oxLDL vs. vehicle (Veh) for 48 h (*n* = 6 for each condition; GEO ID: GSE54666; one-way ANOVA, cutoff, *P* < 0.05). Representative genes encoding major components (log2, one-way ANOVA). (**c–e**) Activation of the NF-κB and HIF pathways. RAW264.7 cells exposed to 25**–**100 μg/mL oxLDL for 2 h **(c)** or 50 μg/mL oxLDL for 0.5-2 h **(d)**; RAW264.7 cells and THP-1-derived macrophages exposed to 50 μg/mL nLDL or oxLDL for 24 h **(e)**. Blots of total and phosphorylated p65 were quantified using ImageJ software. The results of a representative experiment are shown; two additional experiments yielded equivalent results (statistical analysis of quantification data is shown in Supplementary Figure S4).

### 3.5 NF-κB acts as an upstream regulator of the HIF pathway and metabolic rewiring

KEGG PathwayFinder verified that oxLDL activated the NF-κB cascade and its downstream targets, manifested by the upregulation of core signaling components and downstream pro-inflammatory factors (Figure 5a). IKK-selective inhibitor parthenolide (10 μM) pretreatment blocked IKKα/β phosphorylation (27) and simultaneously eliminated oxLDL-induced HIF-1α/1β upregulation and iNOS protein expression (Figure 5b). To exclude off-target drug effects, another specific IKK2 inhibitor IV was used for reciprocal verification, and it reproduced identical pathway suppression (Supplementary Figure S5a). Pharmacological NF-κB inhibition significantly suppressed M1 inflammatory transcription (marked reduction of pro-inflammatory genes), increased M2 marker gene expression (Figures 5c,d), and prevented oxLDL-induced morphological changes in macrophages (Figure 5e; Supplementary Figure S5b). *Ex vivo* assays on 12 atherosclerosis patient-derived primary macrophages yielded consistent phenotypic results (Figure 5f).

**Figure 5:**
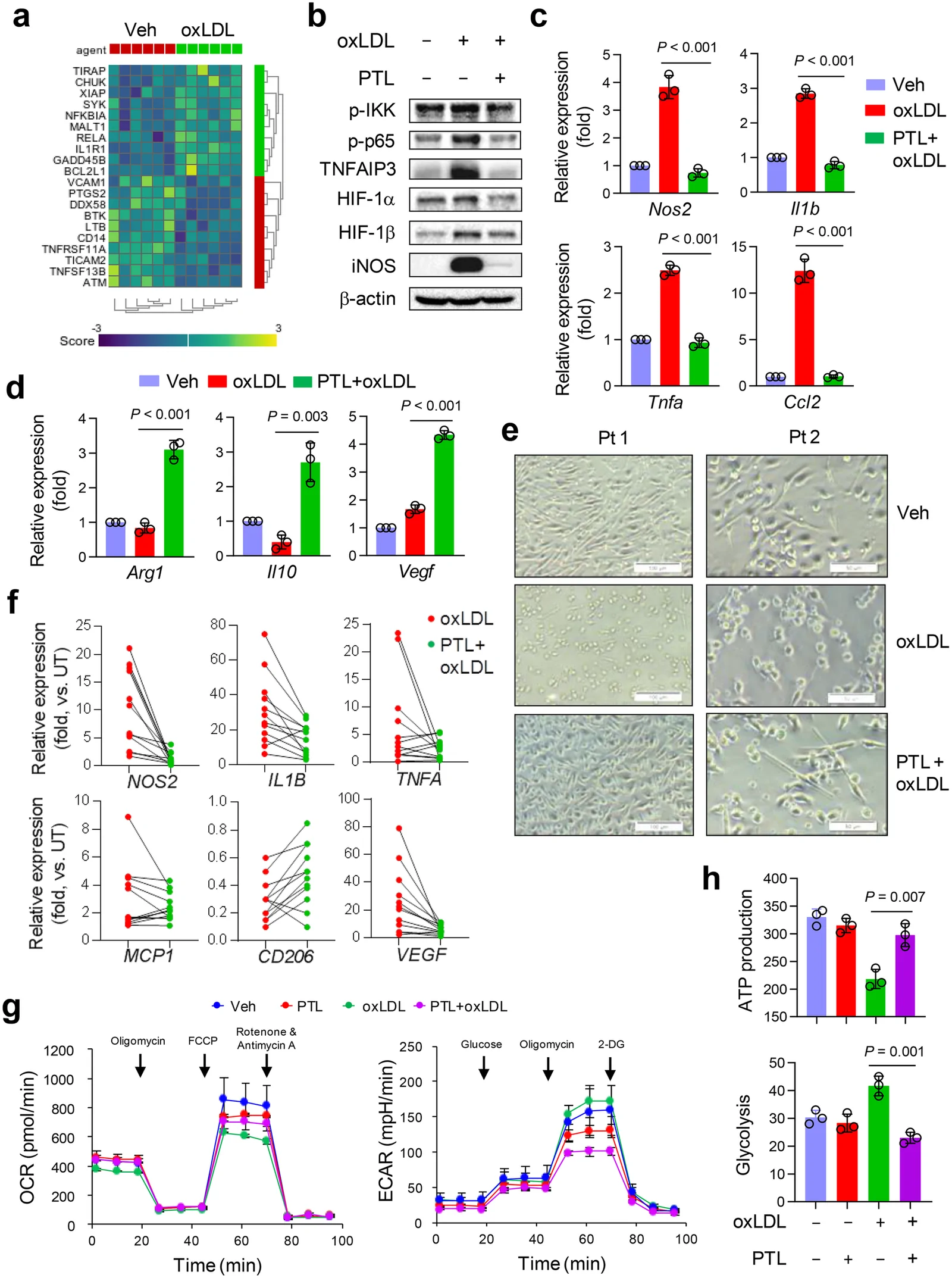
NF-κB inhibition blocks oxLDL-induced macrophage polarization by preventing HIF activation and metabolic shift. **(a)** PathwayFinder analysis (one-way ANOVA, cutoff, *P* < 0.05) of the NF-κB pathway in primary human monocyte-differentiated macrophages exposed to 50 μg/mL oxLDL vs. vehicle (Veh) for 48 h (*n* = 6 for each condition; GEO ID: GSE54666). **(b–d)** Analysis of the NF-κB and HIF pathways [**(b)** the results of a representative experiment are shown; an additional study yielded equivalent results] or M1 **(c)** and M2 gene expression **(d)** in RAW264.7 cells pre-treated with 10 μM PTL for 2 h, followed by 50 μg/mL oxLDL for an additional 24 h. Values represent the mean ± SD for at least three independent experiments (two-tailed Student's *t*-test). For each experiment, the readout of vehicle treatment as a control is arbitrarily set to 1.0. **(e,f)** Representative morphological alterations [**(e)** scale ba*r* = 100 μm and 50 μm for Pt 1 and 2, respectively] and expression of M1 and M2 genes **(f)** in macrophages differentiated from primary monocytes of patients with CHD/IS (*n* = 12) after incubation with PTL ± oxLDL (10 μg/mL). **(g,h)** Example of OCR and ECAR in THP-1-derived macrophages incubated with PTL ± oxLDL **(g)**. Average values of mitochondrial ATP production and glycolysis. Values represent the mean ± SD for at least three independent experiments **(h)**.

After oxLDL co-treatment, NF-κB inhibition restored mitochondrial OXPHOS-dependent ATP production and blocked oxLDL-induced glycolysis enhancement (Figure 5g). Quantification from three independent replicates demonstrated that parthenolide pretreatment restored mitochondrial OXPHOS-dependent ATP production and robustly prevented oxLDL-stimulated glycolytic upregulation (Figure 5h). This demonstrated that NF-κB is located strictly upstream of HIF signaling, governing oxLDL-triggered macrophage inflammatory polarization and metabolic switch.

### 3.6 HIF-1β bridges NF-κB and HIF pathways to mediate pathogenic reprogramming

KEGG PathwayFinder analysis showed that HIF-1β-related upregulated DEGs included *HIF1A*/HIF-1*α*, *HK2* (glycolytic enzymes), *IL6R* (IL-6 receptor), and *VEGFA* (Figure 6a). However, HIF-1β-associated downregulated transcripts were the most strongly enriched in OXPHOS, with weaker enrichment in the TCA cycle (Figure 6b), involving all five mitochondrial electron transport chain complexes (I–V; Supplementary Figure S5c). Two independent *Arnt*-targeting shRNAs achieved high HIF-1β knockdown efficiencies (Figure 6c). HIF-1β deficiency slightly elevated basal IKK and p65 phosphorylation as intracellular compensatory feedback, but it clearly blocked oxLDL-induced HIF-1α stabilization and the elevation of HK2, and iNOS without inhibiting the upstream NF-κB activation cascade (Figure 6c). This clarified that HIF-1β acts as a downstream effector rather than an upstream regulator of NF-κB. Functional verification showed that HIF-1β silencing reduced M1 inflammatory gene expression and elevated M2 anti-inflammatory gene transcription (Figures 6d,e), ultimately preventing oxLDL-induced pro-atherogenic polarization.

**Figure 6:**
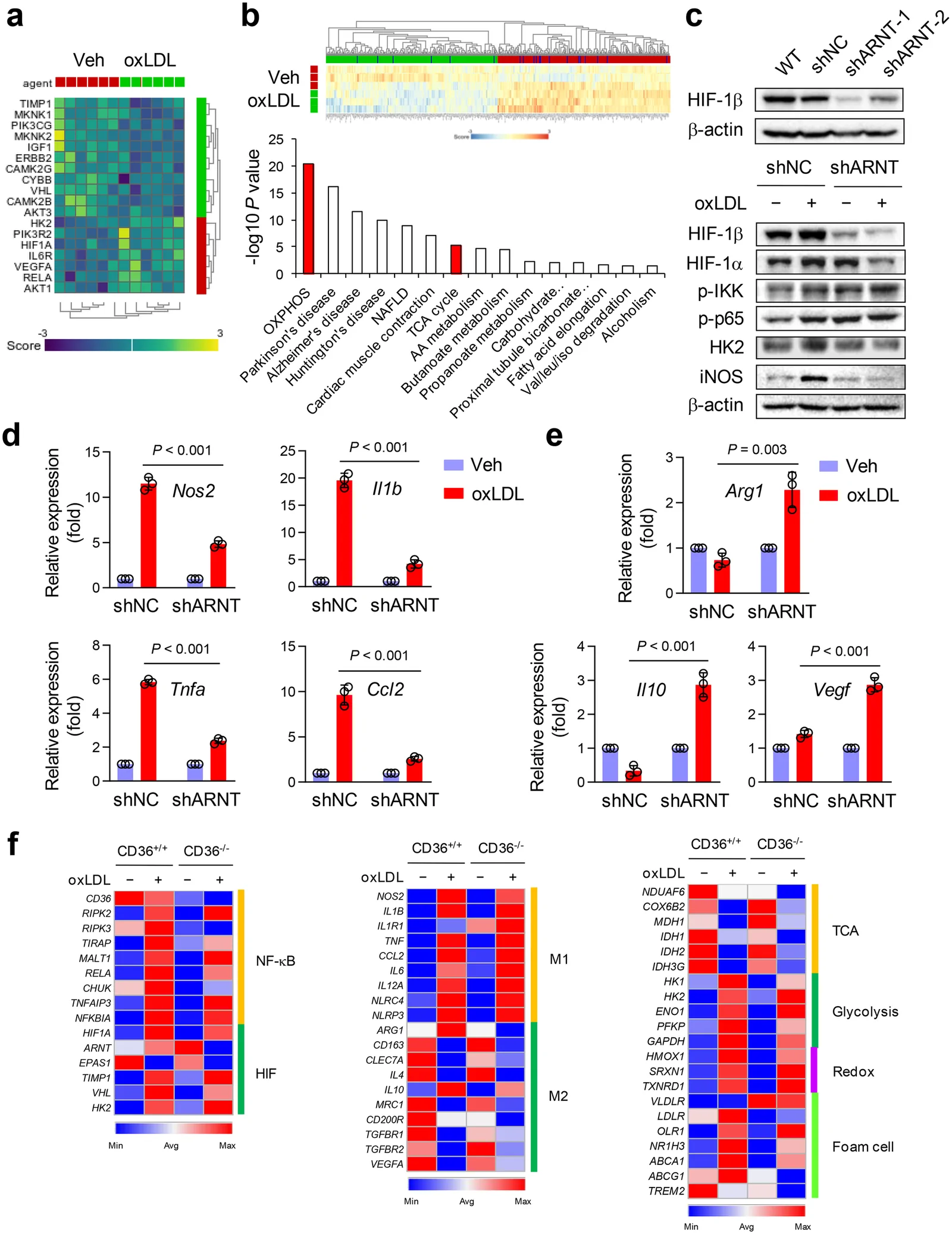
HIF-1β knockdown prevents macrophage polarization without affecting NF-κB activation. **(a)** PathwayFinder analysis (one-way ANOVA, cutoff, *P* < 0.05) of the HIF pathway in primary human monocyte-differentiated macrophages exposed to 50 μg/mL oxLDL vs. vehicle (Veh) for 48 h (*n* = 6 for each condition; GEO ID: GSE54666). **(b)** Expression of HIF-1β-related genes (upper; one-way ANOVA, cutoff, *P* < 0.05) and KEGG analysis (lower; *t*-test, cutoff, *P* < 0.0001). **(c–e)** Effect of *ARNT* knockdown on the NF-κB and HIF pathways, HK2 and iNOS [**(c)** the results of a representative experiment are shown; an additional study yielded equivalent results], and M1 **(d)** vs. M2 **(e)** gene expression after 24-h exposure to 50 μg/mL oxLDL in RAW264.7 cells. Values represent the mean ± SD for at least three independent experiments (two-tailed Student's *t*-test). For each experiment, the readout of vehicle treatment as a control is arbitrarily set to 1.0. **(f)** RNAseq analysis of peritoneal macrophages from wild-type (CD36^+/+^) and CD36-knockout (CD36^−/−^) mice (GEO ID: GSE139439; log2, fold change), after treatment with 20 μg/mL oxLDL for 8 h.

Using an independent public macrophage transcriptome dataset (GSE139439), we validated the phenotypic features of OAMs in murine wild-type macrophages exposed to oxLDL, including pro-inflammatory (along with impaired resolution) and foam cell-forming capacities, simultaneous activation of NF-κB and HIF signaling pathways, and metabolic shift from OXPHOS to glycolysis (Figure 6f). However, CD36 knockout (CD36^−/−^) failed to block oxLDL-induced NF-κB/HIF activation, the core inflammatory signaling cascade, and the glycolytic metabolic shift (Figure 6f). Differential gene analysis confirmed that CD36 deletion only altered lipid trafficking-related transcripts, including *VLDLR*, *LDLR*, *ABCG1*, and foam cell marker *TREM2*, consistent with the function of CD36 as a scavenger receptor mediating foam cell biogenesis (11). These observations suggest that oxLDL activates pathogenic inflammatory and metabolic signaling independent of CD36, while CD36 selectively mediates lipid internalization and foam cell formation, separating two mutually independent pathogenic programs of oxLDL.

### 3.7 Targeting the NF-κB/HIF-1β axis ameliorates macrophage pro-atherogenic dysfunction

OxLDL pretreatment amplified PMA-nigericin-induced NLRP3 inflammasome priming and activation (28), which was manifested as ASC oligomerization, NLRP3-ASC-caspase-1 complex assembly, and caspase-1 cleavage, further boosting macrophage pyroptosis, as reflected by increased GSDMD cleavage, a conventional marker of pyroptosis (Figures 7a–c) (29, 30). Pharmacological NF-κB inhibition reduced inflammasome assembly efficiency, blocked downstream pyroptotic protein cleavage (Figures 7a–c), and eliminated oxLDL-induced priming for a hyper-inflammatory response upon secondary LPS stimulation (Supplementary Figure S5d) (13, 14). In addition, oxLDL enhanced macrophage efferocytosis, and NF-κB/HIF-1β blockade only induced slight efferocytosis reduction (Figure 7d), confirming that this vascular-protective function is largely NF-κB-independent (31). Oil Red O staining confirmed that parthenolide pretreatment inhibited oxLDL-induced intracellular lipid deposition (Figure 7e), effectively arresting foam cell maturation. Collectively, blocking the NF-κB/HIF-1β cascade improves multiple pro-atherogenic macrophage dysfunctions without sacrificing the partial vascular-protective phenotype.

**Figure 7:**
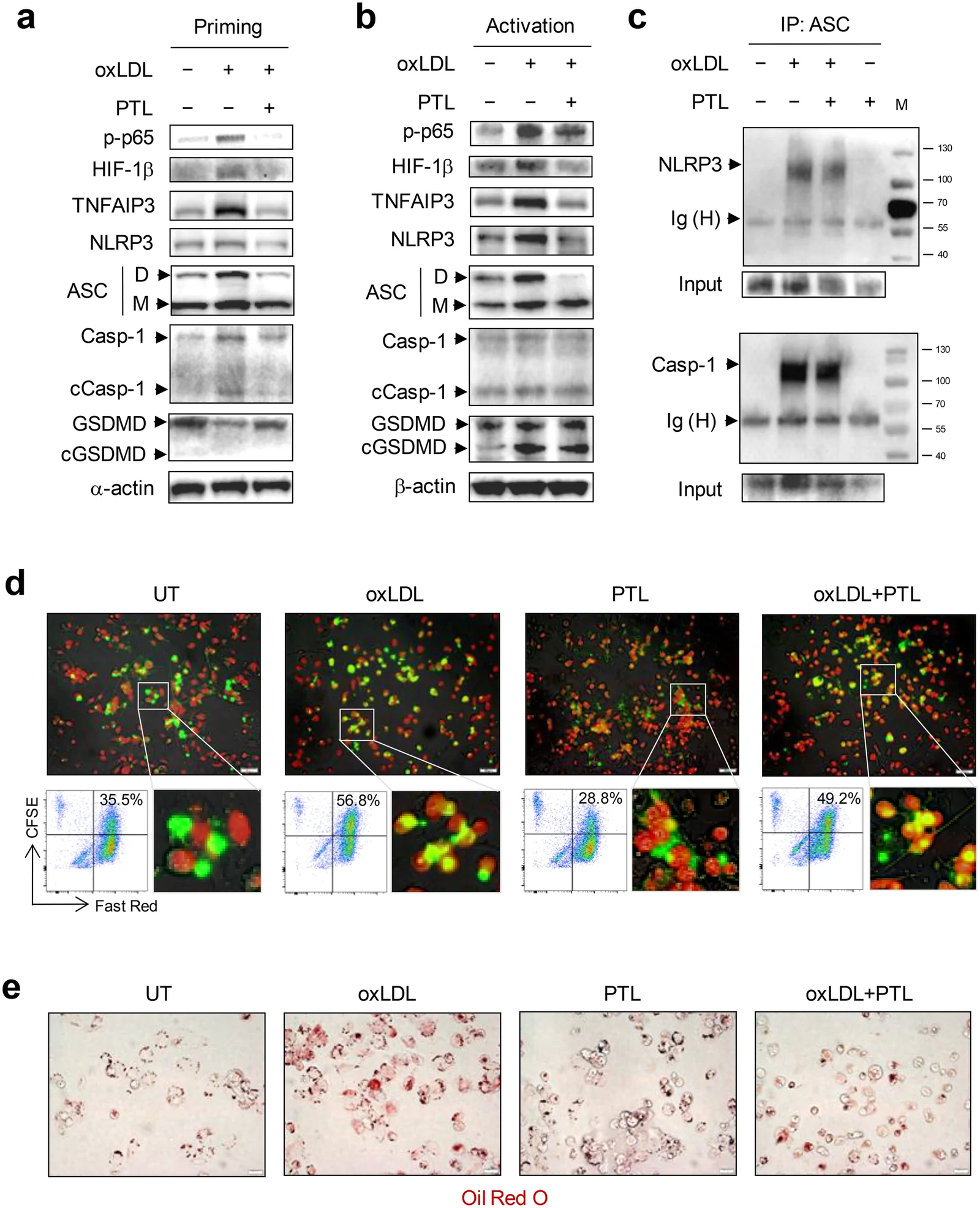
NF-κB inhibition impairs inflammasome, efferocytosis, and lipid uptake. **(a–c)** Analysis of inflammasomes in THP-1 cells exposed to 10 μM PTL ± 50 μg/mL oxLDL during priming or activation. M, monomer; D, dimer; cCasp-1, cleaved caspase-1; cGSDMD, cleaved GSDMD; Ig(H), immunoglobulin heavy chain. The results of a representative experiment are shown, and an additional study yielded equivalent results. **(d)** Example of efferocytosis in THP-1-derived macrophages (Far-Red, red) using apoptotic Jurkat cells (CFSE, green). **(e)** Example of Oil Red O staining for lipid uptake (scale bar = 50 μm).

## 4 Discussion

The limited clinical benefits of existing anti-inflammatory therapies for atherosclerosis, including colchicine-based anti-inflammatory regimens and broad-spectrum cytokine inhibitors, imply the necessity to excavate cell-specific pathogenic mechanisms and precise signaling targets (32). Most prior clinical trials targeted pan-vascular inflammation, ignoring macrophage subset heterogeneity, which leads to offset therapeutic efficacy and adverse immune suppression side effects (2). Therefore, targeting lesional macrophage-specific regulatory pathways is regarded as a next-generation anti-atherosclerotic intervention strategy. Using two independent transcriptomic datasets, encompassing human and murine macrophages exposed to oxLDL (11, 15), this study identified and validated oxLDL-induced OAMs, an atherosclerotic macrophage subset integrating core molecular and functional characteristics of plaque pro-inflammatory and TREM2^hi^ foamy macrophages captured by clinical scRNA-seq datasets (4, 5). OAMs also share certain redox-regulatory genes with Mox, such as *SRXN1* and *TXNRD1*, but not *HMOX1*, the major marker of this macrophage phenotype (24, 33). Distinct from the simplified classical M1/M2 dichotomy and other well-documented plaque macrophage subgroups (M4, Mox, and Mhem) (20, 21, 30), OAMs possess notable transcriptional hallmarks: elevated inflammatory receptors rather than pro-inflammatory cytokines, persistently suppressed inflammation-resolving signals (34, 35), and intrinsic lipid deposition tendency (4). This transcriptional pattern reconciles the conflicting conclusions in previous lipid inflammation studies (36, 37). Multiple studies reported negligible acute pro-inflammatory cytokine secretion upon oxLDL stimulation, while clinical samples still presented aggravated vascular inflammation, which can be explained by enhanced inflammatory sensitization mediated by OAM cytokine receptors (13). Mechanistically, this work clarified a novel normoxia-activated signaling cascade (Figure 8): oxLDL triggers early NF-κB activation, upregulates downstream HIF-1β, and further stabilizes HIF-1α protein independent of hypoxic induction, triggering synergistic transcriptional reprogramming and glycolytic metabolic rewiring to form pathogenic OAMs. In contrast to canonical hypoxia-adaptive HIF activation, oxLDL-driven HIF cascade relies on NF-κB signal and epigenetic priming via H3K27ac chromatin modification, which also provides a novel explanation for sustained inflammation in the normoxic plaque microenvironment.

**Figure 8:**
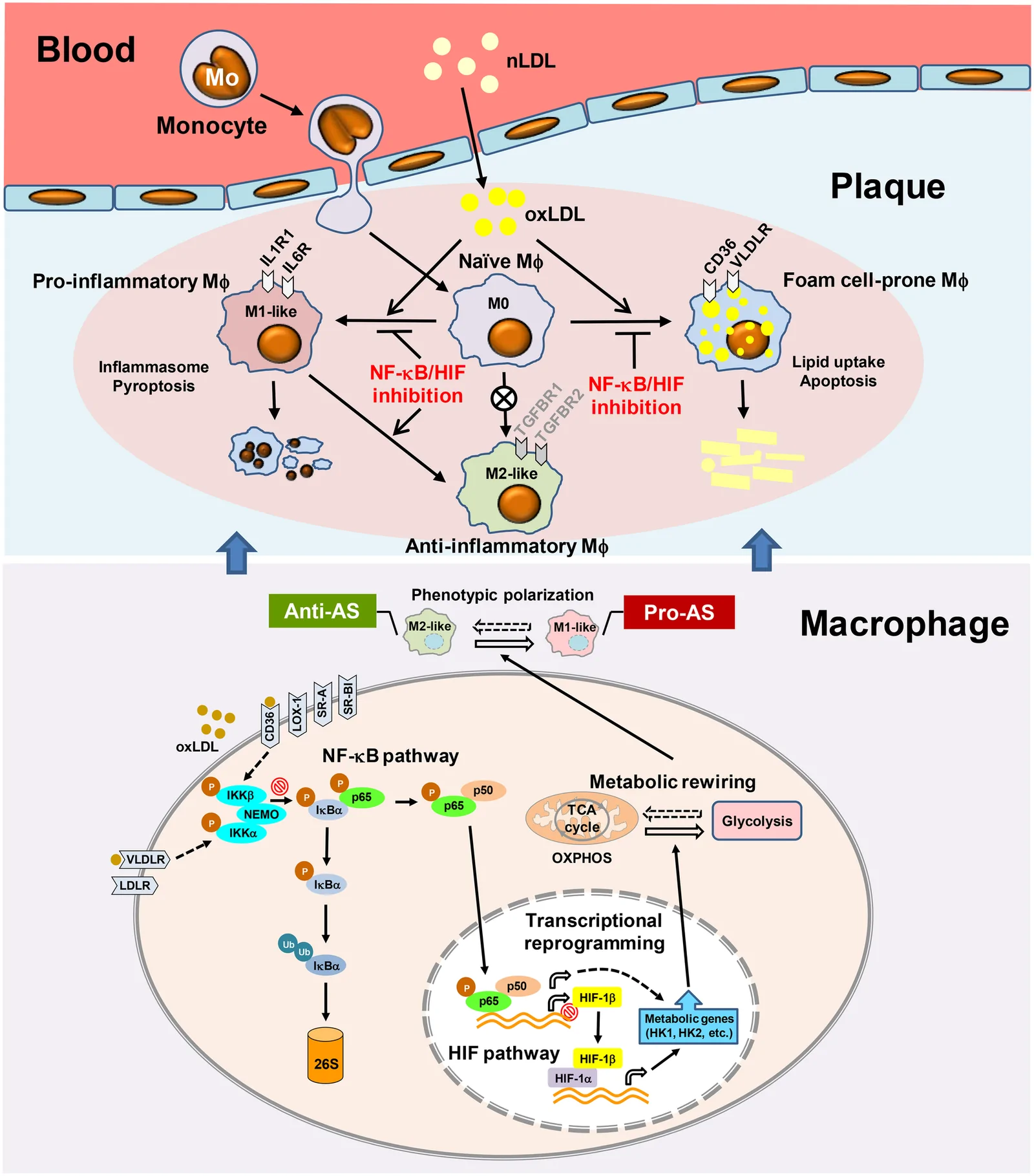
Schematic summary of the proposed mechanism by which oxLDL induces pro-atherogenic polarization of macrophages. Within atherosclerotic lesions, oxLDL drives transcriptional reprogramming in macrophages through metabolic rewiring, shifting cellular bioenergetics from oxidative phosphorylation to glycolysis, and skewing these cells toward a pro-inflammatory, foam cell-prone phenotype. This regulatory cascade relies on NF-κB-dependent upregulation of HIF-1β, revealing a critical signaling crosstalk between the NF-κB and HIF axes.

Consistent with clinical atherosclerotic specimens, hyperlipidemic patients enrolled in this study showed a significant positive correlation between oxLDL-related blood lipid indicators and systemic pro-inflammatory cytokines, consolidating the clinical relevance of the OAM pathogenic mechanism. Functionally, upregulated *IL1R1* and *IL6R* endow OAMs with persistent inflammatory hypersensitivity, which is the essential molecular basis for oxLDL-induced persistent inflammation (13, 14). This epigenetic immune memory enables macrophages to amplify the inflammatory response upon secondary inflammatory stimulation (15, 16), explaining long-term plaque progression even after blood lipid correction in atherosclerosis patients (32). Notably, brief exposure of monocytes to a low concentration of oxLDL induced a long-lasting pro-atherogenic macrophage phenotype, characterized by increased production of pro-inflammatory cytokines and formation of foam cells through epigenetic reprogramming (38). Consistently, our results identified a dual regulatory effect of oxLDL on macrophage functions (pro-inflammatory and foam cell-prone), along with activation of inflammasomes and pyroptosis, as well as moderately enhanced efferocytosis capacity, an endogenous vascular-protective response (31). This paradoxical phenotype partially accounts for plaque heterogeneity in clinical pathology: partial early atherosclerotic lesions spontaneously regress, while advanced lesions progress rapidly (34, 39). More importantly, blocking NF-κB/HIF-1β barely impairs oxLDL-induced efferocytosis, which prevents excessive inhibition of vascular-protective macrophage function during targeted intervention, thereby improving the clinical translational safety of this therapeutic target (32). Meanwhile, oxLDL triggers macrophage pyroptosis, complementing the existing knowledge of plaque macrophage cell death and explaining excessive inflammatory infiltration in ruptured atherosclerotic plaques (40, 41). Moreover, given the well-established role of the NF-κB pathway in regulating the expression of chemokine/adhesion genes, targeting NF-κB may also impair macrophage recruitment into atherosclerotic lesions, thereby reducing the infiltration of CD68^+^ macrophages in plaques.

Previous transcriptomic studies have confirmed that multiple lineage-determining and signal-dependent transcription factors participate in macrophage polarization during atherogenesis (42). The lipid-sensing PPAR family and redox-sensitive Nrf2 signals are universally validated as vascular-protective regulators, which antagonize M1 polarization and alleviate plaque lipid deposition (33). The inconsistent pathogenic phenotype between OAMs and PPAR/Nrf2-activated macrophage subsets further excludes these two pathways as dominant drivers of oxLDL-induced polarization, narrowing the core regulatory signals to inflammatory transcription cascades. In this context, Nrf2 mediates the formation of Mox macrophages, marked by high expression of the antioxidant enzyme HO-1 (encoded by *HMOX*), although their role in atherosclerotic initiation and progression remains uncertain (24). This study verified that HIF-1β is an indispensable crosstalk molecule linking the NF-κB and HIF pathways under normoxia (43, 44). Conventionally, HIF-1β is constitutively expressed (45). We found that oxLDL significantly upregulated HIF-1β expression, especially at the protein level, through an NF-κB-dependent mechanism, alongside the enrichment of H3K27ac signal at enhancer regions of *ARNT*. However, it remains uncertain whether NF-κB directly activates *ARNT* transcription by binding p65 to its promoter or enhancer. Nevertheless, HIF-1β accumulation locks NF-κB-mediated inflammatory transcription and glycolytic metabolic state, forming a positive feedback loop to sustain macrophage pathogenic reprogramming (46, 47). Although HIF-2α exerts protective M2 polarization effects in vascular macrophages (45), HIF-1α-dominated downstream signaling overrides HIF-2α protection under oxLDL stimulation, ultimately determining the pro-atherogenic fate of OAMs. This antagonistic effect between HIF isoforms also provides a new direction for isoform-specific targeted therapy for atherosclerosis.

Metabolic rewiring is the core executor that translates transcription factor activation into macrophage pathogenic phenotypes (22, 25). For instance, LPS exposure increases the levels of succinate by switching the core metabolism from OXPHOS to glycolysis, which in turn stabilizes HIF-1*α*, with IL-1β as a major downstream target (48). Consistent with the classic immunometabolism theory, the oxLDL-induced glycolytic shift is sufficient to sustain persistent NF-κB activation, forming an inflammatory-metabolic positive feedback loop to facilitate plaque deterioration. In contrast to LPS-induced acute glycolysis (13), oxLDL-triggered metabolic rewiring is long-lasting and dependent on HIF-1β transcriptional memory, which is the key metabolic mechanism of persistent inflammation (14). Notably, this study suggested two independent oxLDL pathogenic branches by CD36-knockout reanalysis (36): the CD36-independent NF-κB/HIF-1β inflammatory-metabolic cascade governs macrophage polarization, whereas CD36-dependent lipid uptake signaling selectively mediates the formation of foam cells (49). However, because this two-branch model rests solely on a single external CD36-knockout database under different conditions (including species, oxLDL concentration, and exposure time), it remains to be validated in human atherosclerotic disease. Although nLDL triggers mild NF-κB/HIF activation and metabolic disturbance, proving that native LDL has intrinsic vascular toxicity independent of oxidation modification (22), lipid oxidation dramatically amplifies pathway activation intensity and immune memory durability (12, 14), which explains why hyperlipidemia combined with oxidative stress dramatically increases cardiovascular event risk in clinical cohorts (50–52). In addition, ROS can directly drive pro-inflammatory (M1) polarization of macrophages, at least in part, through the NF-κB and HIF pathways (6). Therefore, targeting the NF-κB/HIF axis can simultaneously block dual pathogenic branches (i.e., inflammation and foam cell formation), achieving synergistic anti-atherosclerotic efficacy superior to single lipid-lowering therapy.

This study still had several limitations. First, although our findings on pro-inflammatory macrophage polarization and the correlation between oxLDL and inflammation were confirmed in atherosclerotic patient samples, core mechanism exploration was largely based on *in vitro* experiments, lacking *in situ* spatiotemporal verification of NF-κB/HIF-1β activation in atherosclerotic plaque tissues. Second, we validated the macrophage-intrinsic regulatory mechanism, although HIF-1β may exert different biological functions in other vascular cell types, such as endothelial cells, as observed in our previous study (44). Third, patient-derived primary macrophage experiments adopted an *ex vivo* stimulation system, which cannot fully simulate the complex blood lipid, inflammatory, and hypoxic microenvironments of human atherosclerotic lesions. Moreover, because the patients enrolled in this study had been receiving statins, antiplatelet agents, and/or other therapies at the time of sampling, their potential influence on the analyses or experiments using primary samples could not be excluded. In future research, we will construct lipid-induced atherosclerotic models using lineage-specific HIF-1β knockout mice for *in vivo* verification, combined with clinical plaque single-cell analyses to validate pathway activation specificity, further consolidating the translational application value of this target. Nevertheless, this study offers a new avenue for exploring how oxLDL fuels atherosclerotic progression.

In conclusion, this study characterized oxLDL-induced OAMs with combined pro-inflammatory and foamy macrophage signatures in atherosclerosis. Mechanistically, HIF-1β acts as a signaling hub to connect the NF-κB and HIF pathways, coordinating macrophage transcriptional reprogramming and the OXPHOS-glycolysis metabolic switch to drive pro-atherogenic polarization. Targeting NF-κB/HIF-1β signaling suppresses macrophage inflammation, prevents metabolic disturbances, and inhibits foam cell formation. This axis is a promising therapeutic target for atherosclerotic vascular disease. Further *in vivo* animal verification and clinical translational research are required to validate its clinical application.

## Data Availability

The original contributions presented in this study are included in the article/Supplementary Material. Further inquiries can be directed to the corresponding authors.

## References

[1] Peter L. The changing landscape of atherosclerosis. Nature. (2021) 592:524–33. 10.1038/s41586-021-03392-8 33883728

[2] Bäck M Yurdagul A Jr Tabas I Öörni K Kovanen PT. Inflammation and its resolution in atherosclerosis: mediators and therapeutic opportunities. Nat Rev Cardiol. (2019) 16:389–406. 10.1038/s41569-019-0169-2 PMC6727648 30846875

[3] Murray PJ. Macrophage polarization. Annu Rev Physiol. (2017) 79:541–66. 10.1146/annurev-physiol-022516-034339 27813830

[4] Willemsen L De Winther MPJ. Macrophage subsets in atherosclerosis as defined by single-cell technologies. J Pathol. (2020) 250:705–14. 10.1002/path.5392 32003464 PMC7217201

[5] Cochain C Vafadarnejad E Arampatzi P Pelisek J Winkels H Ley K. Single-cell RNA-Seq reveals the transcriptional landscape and heterogeneity of aortic macrophages in murine atherosclerosis. Circ Res. (2018) 122:1661–74. 10.1161/CIRCRESAHA.117.312509 29545365

[6] Jimenez-Trinidad FR Morini S Buffon A De Prisco A Galati G De Ciutiis A. Reactive oxygen and nitrogen species on monocyte and macrophage biology. Antioxidants (Basel). (2026) 15:389. 10.3390/antiox15030389 41897534 PMC13023850

[7] Forrester SJ Kikuchi DS Hernandes MS Xu Q Griendling KK. Reactive oxygen species in metabolic and inflammatory signaling. Circ Res. (2018) 122:877–902. 10.1161/CIRCRESAHA.117.311401 29700084 PMC5926825

[8] Borén J Chapman MJ Krauss RM Packard CJ Bentzon JF Binder CJ. Low-density lipoproteins cause atherosclerotic cardiovascular disease: pathophysiological, genetic, and therapeutic insights: a consensus statement from the European atherosclerosis society consensus panel. Eur Heart J. (2020) 41:2313–30. 10.1093/eurheartj/ehz962 PMC7308544 32052833

[9] Hong CG Florida E Li H Parel PM Mehta NN Alexander V. Oxidized low-density lipoprotein associates with cardiovascular disease by a vicious cycle of atherosclerosis and inflammation: a systematic review and meta-analysis. Front Cardiovasc Med. (2023) 9:1023651. 10.3389/fcvm.2022.1023651 36727024 PMC9885196

[10] Babakr AT. Oxidized low-density lipoproteins and their contribution to atherosclerosis. Explor Cardiol. (2025) 3:101246. 10.37349/ec.2025.101246

[11] Chen Y Yang M Huang W Chen W Zhao Y Schulte ML. Mitochondrial metabolic reprogramming by CD36 signaling drives macrophage inflammatory responses. Circ Res. (2019) 125:1087–102. 10.1161/CIRCRESAHA.119.315833 31625810 PMC6921463

[12] Que X Hung M-Y Yeang C Gonen A Prohaska TA Sun X. Oxidized phospholipids are proinflammatory and proatherogenic in hypercholesterolemic mice. Nature. (2018) 558:301–6. 10.1038/s41586-018-0198-8 29875409 PMC6033669

[13] Leentjens J Bekkering S Joosten LAB Netea MG Burgner DP Riksen NP. Trained innate immunity as a novel mechanism linking infection and the development of atherosclerosis. Circ Res. (2018) 122:664–9. 10.1161/CIRCRESAHA.117.312465 29367213

[14] Keating ST Groh L Thiem K Bekkering S Li Y Matzaraki V. Rewiring of glucose metabolism defines trained immunity induced by oxidized low-density lipoprotein. J Mol Med. (2020) 98:819–31. 10.1007/s00109-020-01915-w 32350546 PMC7297856

[15] Reschen ME Gaulton KJ Lin D Soilleux EJ Morris AJ Smyth SS. Lipid-induced epigenomic changes in human macrophages identify a coronary artery disease-associated variant that regulates PPAP2B expression through altered C/EBPBeta binding. PLoS Genet. (2015) 11:e1005061. 10.1371/journal.pgen.1005061 25835000 PMC4383549

[16] Kuznetsova T Prange KHM Glass CK De Winther MPJ. Transcriptional and epigenetic regulation of macrophages in atherosclerosis. Nat Rev Cardiol. (2020) 17:216–28. 10.1038/s41569-019-0265-3 31578516 PMC7770754

[17] Jin F Li J Guo J Doeppner TR Hermann DM Yao G. Targeting epigenetic modifiers to reprogramme macrophages in non-resolving inflammation-driven atherosclerosis. Eur Heart J Open. (2021) 1:oeab022. 10.1093/ehjopen/oeab022 35919269 PMC9241575

[18] Duncan JA Canna SW. The NLRC4 inflammasome. Immunol Rev. (2018) 81:115–23. 10.1111/imr.12607 PMC5897049 29247997

[19] Yang X Cong T He H Wang J. GSDME maintains hematopoietic stem cells by balancing pyroptosis and apoptosis. Blood Sci. (2020) 3:40–7. 10.1097/BS9.0000000000000064 35402833 PMC8975053

[20] Lancaster GI Langley KG Berglund NA Kammoun HL Reibe S Estevez E. Evidence that TLR4 is not a receptor for saturated fatty acids but mediates lipid-induced inflammation by reprogramming macrophage metabolism. Cell Metab. (2018) 27:1096–110. 10.1016/j.cmet.2018.03.014 29681442

[21] Barrett TJ. Macrophages in atherosclerosis regression. Arterioscler Thromb Vasc Biol. (2020) 40:20–33. 10.1161/ATVBAHA.119.312802 31722535 PMC6946104

[22] Lee J Choi J-H. Deciphering macrophage phenotypes upon lipid uptake and atherosclerosis. Immune Netw. (2020) 20:e22. 10.4110/in.2020.20.e22 32655970 PMC7327152

[23] Jinnouchi H Guo L Sakamoto A Torii S Sato Y Cornelissen A. Diversity of macrophage phenotypes and responses in atherosclerosis. Cell Mol Life Sci. (2020) 77:1919–32. 10.1007/s00018-019-03371-3 31720740 PMC11104939

[24] Kadl A Meher AK Sharma PR Lee MY Doran AC Johnstone SR. Identification of a novel macrophage phenotype that develops in response to atherogenic phospholipids via Nrf2. Circ Res. (2010) 107:737–46. 10.1161/CIRCRESAHA.109.215715 20651288 PMC2941538

[25] Zhang D Tang Z Huang H Zhou G Cui C Weng Y. Metabolic regulation of gene expression by histone lactylation. Nature. (2019) 574:575–80. 10.1038/s41586-019-1678-1 31645732 PMC6818755

[26] Lin N Simon MC. Hypoxia-inducible factors: key regulators of myeloid cells during inflammation. J Clin Invest. (2016) 126:3661–71. 10.1172/JCI84426 27599290 PMC5096831

[27] Dai Y Guzman ML Chen S Wang L Yeung S-K Pei X-Y. The NF (nuclear factor)-(B inhibitor parthenolide interacts with histone deacetylase inhibitors to induce MKK7/JNK1-dependent apoptosis in human acute myeloid leukaemia cells. Br J Haematol. (2010) 151:70–83. 10.1111/j.1365-2141.2010.08319.x 20701602 PMC2950247

[28] Glass CK Natoli G. Molecular control of activation and priming in macrophages. Nat Immunol. (2015) 17:26–33. 10.1038/ni.3306 PMC4795476 26681459

[29] Lugrin J Martinon F. Detection of ASC oligomerization by western blotting. Bio Protoc. (2017) 7:e2292. 10.21769/BioProtoc.2292 29085861 PMC5660620

[30] Demarco B Grayczyk JP Bjanes E Le Roy D Tonnus W Assenmacher C-A. Caspase-8–dependent gasdermin D cleavage promotes antimicrobial defense but confers susceptibility to TNF-induced lethality. Sci Adv. (2020) 6:eabc3465. 10.1126/sciadv.abc3465 33208362 PMC7673803

[31] Kojima Y Volkmer J-P McKenna K Civelek M Lusis AJ Miller CL. CD47-blocking antibodies restore phagocytosis and prevent atherosclerosis. Nature. (2016) 536:86–90. 10.1038/nature18935 27437576 PMC4980260

[32] Libby P Everett B. Novel anti-atherosclerotic therapies. Arterioscler Thromb Vasc Biol. (2019) 39:538–45. 10.1161/ATVBAHA.118.310958 30816799 PMC6436984

[33] Harada N Ito K Hosoya T Mimura J Maruyama A Noguchi N. Nrf2 in bone marrow-derived cells positively contributes to the advanced stage of atherosclerotic plaque formation. Free Radic Biol Med. (2012) 53:2256–62. 10.1016/j.freeradbiomed.2012.10.001 23051009

[34] Rahman K Vengrenyuk Y Ramsey SA Vila NR Girgis NM Liu J. Inflammatory Ly6Chi monocytes and their conversion to M2 macrophages drive atherosclerosis regression. J Clin Invest. (2017) 127:2904–15. 10.1172/JCI75005 28650342 PMC5531402

[35] Kasikara C Doran AC Cai B Tabas I. The role of non-resolving inflammation in atherosclerosis. J Clin Invest. (2018) 128:2713–23. 10.1172/JCI97950 30108191 PMC6025992

[36] Sheedy FJ Grebe A Rayner KJ Kalantari P Ramkhelawon B Carpenter SB. CD36 coordinates NLRP3 inflammasome activation by facilitating intracellular nucleation of soluble ligands into particulate ligands in sterile inflammation. Nat Immunol. (2013) 14:812–20. 10.1038/ni.2639 23812099 PMC3720827

[37] Christ A Günther P Lauterbach MAR Duewell P Biswas D Pelka K. Western diet triggers NLRP3-dependent innate immune reprogramming. Cell. (2018) 172:162–75. 10.1016/j.cell.2017.12.013 29328911 PMC6324559

[38] Bekkering S Quintin J Joosten LA Van Der Meer JW Netea MG Riksen NP. Oxidized low-density lipoprotein induces long-term proinflammatory cytokine production and foam cell formation via epigenetic reprogramming of monocytes. Arterioscler Thromb Vasc Biol. (2014) 34:1731–8. 10.1161/ATVBAHA.114.303887 24903093

[39] Yu H. Atherosclerotic plaque regression: experimental approaches and therapeutic advances. Trends Cell Biol. (2021) 31:424–7. 10.1016/j.tcb.2021.03.003 33726967

[40] Goto J Kinoshita D Otaki Y Ra S Ono H Mito T. Oxidized low-density lipoprotein cholesterol and plaque vulnerability. Am Heart J Plus Cardiol Res Pract. (2026) 61:100691. 10.1016/j.ahjo.2025.100691 PMC12723116 41446700

[41] Hoeksema MA Glass CK. Nature and nurture of tissue-specific macrophage phenotypes. Atherosclerosis. (2019) 281:159–67. 10.1016/j.atherosclerosis.2018.10.005 30343819 PMC6399046

[42] Geng T Yan Y Xu L Cao M Xu Y Pu J. CD137 signaling induces macrophage M2 polarization in atherosclerosis through STAT6/PPAR*δ* pathway. Cell Signal. (2020) 72:109628. 10.1016/j.cellsig.2020.109628 32247042

[43] Rius J Guma M Schachtrup C Akassoglou K Zinkernagel AS Nizet V. NF-kappaB links innate immunity to the hypoxic response through transcriptional regulation of HIF-1alpha. Nature. (2008) 453:807–11. 10.1038/nature06905 18432192 PMC2669289

[44] Jin F Zheng X Yang Y Yao G Ye L Doeppner TR. Impairment of hypoxia-induced angiogenesis by LDL involves a HIF-centered signaling network linking inflammatory TNFα and angiogenic VEGF. Aging. (2019) 11:328–49. 10.18632/aging.101726 30659163 PMC6366960

[45] Watts ER Walmsley SR. Inflammation and hypoxia: HIF and PHD isoform selectivity. Trends Mol Med. (2019) 25:33–46. 10.1016/j.molmed.2018.10.006 30442494

[46] Schito L Rey S. Cell-autonomous metabolic reprogramming in hypoxia. Trends Cell Biol. (2018) 28:128–42. 10.1016/j.tcb.2017.10.006 29191366

[47] Semba H Takeda N Isagawa T Sugiura Y Honda K Wake M. HIF-1alpha-PDK1 axis-induced active glycolysis plays an essential role in macrophage migratory capacity. Nat Commun. (2016) 7:11635. 10.1038/ncomms11635 27189088 PMC4873978

[48] Tannahill GM Curtis AM Adamik J Palsson-McDermott EM McGettrick AF Goel G. Succinate is an inflammatory signal that induces IL-1β through HIF-1α. Nature. (2013) 496:238–42. 10.1038/nature11986 23535595 PMC4031686

[49] Wen S-Y Zhi X Liu H-X Wang X Chen Y-Y Wang L. Is the suppression of CD36 a promising way for atherosclerosis therapy? Biochem Pharmacol. (2024) 219:115965. 10.1016/j.bcp.2023.115965 38043719

[50] Thangasparan S Kamisah Y Ugusma A Anuar NNM Ibrahim N. Unravelling the mechanisms of oxidised low-density lipoprotein in cardiovascular health: current evidence from *in vitro* and *in vivo* studies. Int J Mol Sci. (2024) 25:13292. 10.3390/ijms252413292 39769058 PMC11676878

[51] Bekbossynova M Saliev T Ivanova-Razumova T Andossova S Kali A Myrzakhmetova G. Beyond cholesterol: emerging risk factors in atherosclerosis. J Clin Med. (2025) 14:2352. 10.3390/jcm14072352 40217801 PMC11989677

[52] Al-Kufaishi AMA Al-Musawi NJT. Oxidized low-density lipoprotein and atherosclerosis. J Prev Diagn Treat Strat Med. (2025) 4:77–82. 10.4103/jpdtsm.jpdtsm_30_25

