# Supplementary material for: OxLDL drives pro-inflammatory and foam cell-prone macrophage reprogramming via NF-κB/HIF-1β-dependent metabolic rewiring
Source: Front Cardiovasc Med. 2026 Oct 7;13:1957621. doi: 10.3389/fcvm.2026.1957621 (PMC13683113; doi:10.3389/fcvm.2026.1957621)
Supplement: Supplementary file 1 [file Supplementaryfile1.docx]

**OxLDL drives pro-inflammatory and** **foam cell-prone macrophage reprogramming via NF-κB/HIF-1β-dependent metabolic rewiring**

**Supplemental methods**

**Cells and reagents**

Murine macrophage cell line RAW264.7 and human monocytic cell line THP-1 were obtained from American Type Culture Collection (ATCC; Rockille, MD). RAW264.7 cells were maintained in DMEM medium (Corning, NY, USA) containing 10% heat inactivated fetal bovine serum (FBS; Gibco, Thermo Fisher Scientific, Frederick, MD) and supplemented with 2 mM L-glutamine. THP-1 cells were cultured in RPMI-1640 medium (Corning) containing 10% heat inactivated FBS. For differentiating THP-1 cells into macrophages, THP-1 cells were incubated with 50 ng/ml phorbol 12-myristate 13-acetate (PMA; Sigma-Aldrich, St. Louis, MO) for 48 hrs, followed by incubation with RPMI-1640 medium without PMA for additional 24 hrs (resting) prior to the experiment. Considering the potential effect of PMA on pro-inflammatory gene expression, the observations involving the pro- or anti-inflammatory function of macrophages were further validated in RAW264.7 macrophages.

Human native LDL (nLDL; Cat # H7960) and oxidized LDL (oxLDL; Cat # IO1300) were purchased from Beijing Solarbio Science & Technology Co. Ltd (Beijing, China). Briefly, nLDL was isolated from blood bank produced human plasma and purified to homogeneity via ultra-centrifugation (1.019-1.063g/cc) and membrane (0.22 micron) filtered aseptically in a solution containing PBS (pH 7.4) and 0.01 mM EDTA, which yielded 98% purity of nLDL determined by agarose gel electrophoresis. Purified nLDL was oxidized by incubation with 5 μM Cu_2_SO_4_ in PBS at 37°C for 20 hrs and oxidation was then terminated by adding excess EDTA, followed by dialysis against PBS (pH 7.4) containing 0.2 mM EDTA for 48 hrs to remove copper and membrane (0.22 micron) filtered. Both nLDL and oxLDL were stored at 2-8^o^C and used within six weeks. In all experiments, PBS solution (pH 7.4) containing 0.2 mM EDTA was used as vehicle control. To ensure the comparability with the study that generated the data for the transcriptomic analyses in this study, the same concentration of oxLDL (50 μg/ml) was used in most experiments, unless otherwise indicated. Parthenolide, an IκB kinase (IKK) inhibitor, was purchased from Merck Millipore (Billerca, MA). IKK2 inhibitor IV, nigericin, and lipopolysaccharide (LPS) were obtained from Sigma-Aldrich.

**Patient samples**

This study was approved by the institutional review board of the First Hospital of Jilin University (#2019-055). The investigation conformed to the principles outlined in the Declaration of Helsinki. Samples were obtained with informed consent from patients undergoing routine diagnostic and treatment procedures. Clinical information of the enrolled patients included in this study was summarized in **Supplemental Table S1**.

Peripheral blood samples were obtained from a total of 27 patients with CHD and IS. Mononuclear cells isolated using Ficoll^®^ solution (Sigma-Aldrich) from 18 patients were differentiated into macrophages by incubation with human M-CSF (Cat # 216-MC-005; R & D Systems, Minneapolis, MN). Briefly, 2x10^6^ cells/ml were plated on 12-well plates and incubated for 3 hrs with RPMI-1640 medium containing 10% heat inactivated FBS, 4 mM L-glutamine, 50 units/ml penicillin, and 50 μg /ml streptomycin (Sigma-Aldrich), after which cells were wahsed twice to remove unadherent cells. Adherent cells were further cultured in full medium containing 50 ng/ml M-CSF for 6 days, with medium replacement every two days. M-CSF was removed prior to *ex vivo* experiments.

Serum samples were prepared from peripheral blood of patients with (n = 12) or without hyperlipidemia (n = 9), taken prior to treatment (e.g., embolectomy), and subjected to the analysis of soluble cytokines. All enrolled patients had been receiving statins, antiplatelet agents, and/or other therapies at sampling. Plasma levels of oxLDL in patients with hyperlipidemia (n = 6) were measured by an ELISA-based method using Oxidized Low Density Lipoprotein Detection Kit as per manufacturer’s instruction (Huasheng Biotechnolgy Co. Inc, Chengdu, China). Hyperlipidemia was defined as blood levels of LDL-cholesterol (LDL-c) or triglyceride (TG) higher than the normal ranges (< 2.6 mmol/L or 100 mg/dL for LDL-c; < 1.7 mmol/L or 150 mg/dL for TG). Embolus samples were obtained from patients with IS (n = 4) undergoing routine embolectomy and subjected to HE and IHC staining.

**Short hairpin RNA (shRNA)**

Constructs encoding shRNA specifically targeting *ARNT* (encoding HIF-1β) were prepared using the pGreenpuro vector flagged with green fluorescent protein (GFP), including two shRNAs targeting different sequences of mouse *Arnt* gene (shARNT-1: 5'-CCAACAACTTCTAAGAGAT-3'; shARNT-2: 5'-CCAGACAAGCTAACCATCTTACTC -3') and a control shRNA (shNC) targeting ascrambled sequence. For lentiviral packing, HEK 293T cells were seeded in complete DMEM medium a day before transfection and then transfected with the constructs encoding shARNT-1, shARNT-2, or shNC along with psPAX2 and PMD2G using Lipofectamine 2000 (Invitrogen, Carlsbad, CA). After 6 hrs, transfection medium was replaced with fresh DMEM medium. Viral supernatants were harvested at 24 and 48 hrs post-transfection. RAW264.7 cells were cultured with conditional medium containing viral particles for 48 hrs. Transduction efficiency (> 70%) was determined by monitoring the percentage of GFP-positive cells using flow cytometry. Subsequently, transfected cells were then selected by puromycin. Western blot analysis was performed to confirm down-regulation of HIF-1β.

**Quantitative real-time PCR (qPCR)**

Total RNA was extracted from cells using Easy Pure RNA Kit (Transgene Biotech, Beijing, China) as per manufacturer’s instruction. 1 μg of total RNA per condition was reversely transcribed to cDNA and amplified with SYBR (Roche) by real-time PCR as follows: 95℃ for 10 minutes, followed by 40 cycles of 95℃ for 10 seconds, and then 60℃ for 30 seconds. All PCR reactions were run in triplicate, and gene expression relative to GAPDH was calculated using the 2^-ΔΔCT^ method.

Primers for mouse genes include: *Nos2* forward: 5’-GTTCTCAGGCCAACAATACAAG A-3’, *Nos2* reverse: 5’-GTGGACGGGTCGATGTCAC-3’; *Il1b* forward: 5’-GAAGTTGACG GACCCCAAAA-3’, *Il1b* reverse: 5’-CCACAGCCACAATGAGTGATAC-3’; *Tnfa* forward: 5’-TCGTAGCAAACCACCAAGTG-3’, *Tnfa* reverse: 5’-GGAGTAGACAAGGTACAACCC A-3’; *Mcp1* forward: 5’-GCTCAGCCAGATGCAGTTA-3’, *Mcp1* reverse, 5’-CTGCTGGTG ATCCTCTTGTAG-3’; *Arg1* forward: 5’-ACCTGGCCTTTGTTGATGTC-3’, *Arg1* reverse: 5’-ACTGCCAGACTGTGGTCTCC-3’; *Il10* forward: 5’-CGGGAAGACAATAACTGCACC C-3’, *Il10* reverse: 5’-CGGTTAGCAGTATGTTGTCCAGC-3’; *Vegf* forward: 5’-ACTGGAC CCTGGCTTTACTG-3’, *Vegf* reverse: 5’-TCTGCTCTCCTTCTGTCGTG-3’; *Gapdh* forward: 5’-TGCACCACCAACTGCTTAGC-3’, *Gapdh* reverse: 5’-GGATGCAGGGATGATGTTCT -3’.

Primers for human genes include: *NOS2* forward: 5’-TGCAGACACGTGCGTTACTCC -3’, *NOS2* reverse: 5’- GGTAGCCAGCATAGCGGATG-3’; *IL1B* forward: 5′- CAGAAGTAC CTGAGCTCGCC-3′, *IL1B* reverse: 5′-AGATTCGTAGCTGGATGCCG-3′; *TNFA* forward: 5’-ATCCTGGGGGACCCAATGTA-3’, *TNFA* reverse: 5’-AAAAGAAGGCACAGAGGCCA -3’; *MCP1* forward: 5′- CAGCCAGATGCAATCAATGCC-3′, *MCP1* reverse: 5′-TGGAAT CCTGAACCCACTTCT-3′; *VEGF* forward: 5’-AGGGCAGAATCATCACGAAGT-3’, *VEGF* reverse: 5’- AGGGTCTCGATTGGATGGCA-3’; *CD206* forward: 5’-GGGTTGCTATCACT CTCTATGC-3’, CD206 reverse: 5’-TTTCTTGTCTGTTGCCGTAGTT-3’; *GAPDH* forward: 5’-AGAAGGCTGGGGCTCATTTG-3’, *GAPDH* reverse: 5’-AGGGGCCATCCACAGTCTT C-3’.

**Western blot and immunoprecipitation**

Whole cell lysates were prepared in RIPA lysis buffer (Cell Signalling Technology, Danvers, MA) containing phenylmethylsulphonyl fluoride (PMSF) and phosphatase inhibitors (Roche, Berlin, Germany). Total protein was quantified by using BCA protein assay (Thermo Fisher Scientific). Equal amounts of protein (20 ~ 30 μg) were resolved by sodium dodecyl sulfate polyacrylamide gel electrophoresis (SDS-PAGE), followed by transferring to polyvinylidene fluoride (PVDF) membranes. Membranes were blocked with TBS-Tween 20 containing 5% bovine serum albumin (BSA) at room temperature for 1 hr and then probed with the appropriate dilution of primary antibody overnight at 4^o^C, followed by incubation with a 1:5000 dilution of horseradish peroxidase (HRP)-conjugated secondary antibody (Dingguo, Beijing, China) at room temperature for 1 hr. Blots were visualized using Enhanced Chemiluminescence (ECL) Kit (PerkinElmer, Waltham, MA). Following antibodies were used as primary antibodies: anti-HIF-1α, anti-HIF-1β, anti-phospho-NF-κB p65 (Ser536), anti-NF-κB p65, anti-phospho-IKKα/β (Ser180/S181), anti-IKKα/β, anti-NIK, anti-TNFAIP3, anti-IL-1β, anti-iNOS, and anti-GSDMD antibodies from Cell Signalling Technology (Danvers, MA); anti-TRAF3 antibody from Santa Cruz Biotechnology (Dallas, TX); anti-Caspase-1 (D7F10, rabbit mAb #3866), anti-NLRP3, anti-ASC antibody from AdipoGen Life Science (San Diego, CA). Where indicated, blots were reprobed with β-actin or GAPDH antibody (Cell Signalling Technology) to ensure equal loading and transfer of proteins.

**Seahorse analysis**

To examined intracellular energy metabolism, oxygen consumption rate (OCR) and extracellular acidification rate (ECAR) were measured by Agilent Seahorse XFe24 Analyzer using Seahorse XF Cell Mito Stress Test Kit or Seahorse XF Cell Mito Stress Test Kit as per the manufacturer's instructions (Agilent Technologies Inc., Wilmington, DE). Each experiment was performed in triplicate, from which average values were obtained and used for analyzing ATP production and glycolysis. Each experiment was repeated at least three times.

**Cytometric bead array (CBA)**

Serum levels of soluble cytokines (e.g., TNF-α, IL-1β, IL-6, IL-8, IL-10, and IL-12) in patients were determined by flow cytometry (BD Biosciences, San Jose, CA) using Cytometric Bead Array Kit as per manufacturer’s instruction (BD PharMingen, Franklin Lakes, NJ). Each sample (50μl/patient) was measured in duplicate. The concentrations of cytokines were quantified by CellQuest Pro using CBA software.

**Analysis of lipid uptake**

After incubation with oxLDL for 24 hrs, Oil Red O staining was performed to examine lipid uptake by macrophages. Briefly, cells were fixed with 4% paraformaldehyde for 20 min on ice. After washed with PBS, cells were incubated with propylene glycol for 7 min at room temperature, followed by 0.05% Oil Red O in 85% propylene glycol (Sigma-Aldrich; filtered before use) for 5 min. After washed with PBS, images were captured under light microscopy.

**Analysis of inflammasome**

For induction of inflammasome, THP-1 cells were incubated with 300 ng/ml PMA or 1 μg/ml LPS for 3 hrs (priming), followed by 1 μM nigericin (Cat # tlrl-nig,tlrl-nig-5) for 1 hr (activation) as per manufacturer's instructions (InvivoGen, San Diego, CA). Western blot analysis was performed to monitor NLRP3 expression, ASC dimerization, and caspase-1 cleavage. Alternatively, 100 μg protein per condition was immunoprecipitated by anti-ASC antibody using Invitrogen Dynabeads (ThermoFisher, Waltham, MA), after which Western blot analysis was performed to detect binding of NLRP3 or caspase-1.

**Analysis of efferocytosis**

Briefly, Jurkat cells and THP-1-derived macrophages were pre-stained using living cell fluorescent dyes CellTrace™ CFSE and and CellTrace™ Far Redas per manufacturer’s instruction (Invitrogen), respectively. After induction of apoptosis by 10 nM bortezomib for 24 hrs (~50% of apoptotic cells determined by flow cytometry using Annexin V/ PI staining), Jurkat cells were washed twice and then added to culturing macrophages pre-stained with Far Red. After 24-hr incubation, images were captured under fluorescent microscopy (IX83; Olympus, Tokyo, Japan). Alternatively, flow cytometric analysis was performed to determine the percentage of CFSE and Fast Red double-positive cells, indicating engulfment of apoptotic cells by macrophages (yellow).

**Analysis of sensitivity to secondary stimuli**

Sensitivity to secondary stimuli was analyzed by following the procedure described previously. Briefly, THP-1 cells were incubated with 50 μg/ml oxLDL for 24 hrs, after which cells were washed twice and further incubated in culture medium without oxLDL for 3 days. Cells were then re-stimulated with low dose of LPS (10 ng/ml) for 24 hrs, after which qPCR was performed to determine mRNA levels of the pro-inflammatory cytokine TNF-α and IL-1β.

**Immunohistochemistry (IHC)**

Cryostat section of embolus samples obtained from IS patients via embolectomy were prepared and then subjected to H.E. and IHC staining using anti-CD68 and anti-CD163 antibodies (Invitrogen). After incubation with HRP-conjugated secondary antibodies (Abcam), target proteins were visualized by DAB solution. For each sample, integrated option density (IOD) was measured in three fields per section using Image J software. Average optical density (AOD) was then calculated using the formula AOD = IOD / area.

**Transcriptomic analysis**

Transcriptomic analyses (including K-means clsutering, KEGG, KEGG PathwayFinder, and GO) were conducted using R2: Genomics Analysis and Visualization Platform (http://r2.amc.nl) to survey the genome-wide gene expression profile (GEP) in macrophages (differentiated from primary human monocytes by 50 ng/ml M-CSF) after exposure to 50 μg/ml oxLDL for 48 hrs. The publically available datasets used in this study originated from the SuperSeries GSE54975,[13] encompassing the Subseries GSE54666 (gene expression in primary human macrophages and foam cells) used for transcriptomic analyses and the Subseries GSE54972 (ChIP-seq of H3K27ac in primary human macrophages and foam cells)used for analyzing the enrichment of H3K27Ac at the loci of target genes (e.g., *HIF1A*, *ARNT*, *EPAS1*, *CHUK*, and *RELA*). In addition, an independent dataset GSE139439 (next generation sequencing facilitates quantitative analysis of wild type and CD36^-/-^ murine peritoneal macrophage transcriptomes) was used for validating OAMs and examining the effect of CD36 knockout on oxLDL-induced reprogramming of macrophages.

**Statistical analysis**

Values represent the means ± standard deviation (SD) for at least three independent experiments performed in triplicate. The significance of differences between experimental conditions was determined by two-tailed Student's t-test using the GraphPad Prism version 7.0. One-way analysis of variance (ANOVA) was used to determine the significance of differences between experimental conditions in bioinformatics analyses. *P* < 0.05 is considered as statistically significant.

**Supplemental data**

**Table S1. Clinical information of all patients included in this study**

|  | **Sex (age)** | **Diagnosis** | **Serum level (mmol/L)** | | | | **Treatment** | **Sampling** |
| --- | --- | --- | --- | --- | --- | --- | --- | --- |
|  |  |  | **TG** | **TC** | **LDL-c** | **HDL-c** |  |  |
| **1** | M (83) | CHD, AMI, Killip I, hypertension (III) | 0.98 | 3.96 | 1.86 | 1.49 | Atorvastatin, SM, clexane, aspirin, plavix, spirolactone | MNC^a^  Serum^b^ |
| **2** | M (67) | CHD, AMI, Killip II, AU, IMH, CRF, hypertension (III), DM (type II) | 0.88 | 4.08 | 2.31 | 1.07 | Atorvastatin, metoprolol, Norvasc, sosorbide mononitrate, aspirin, plavix, clexane, micardis, Haikun Shenxi, Niaoduqing, nifedipine, sulbenicillin | MNC^a^  Serum^b^ |
| **3** | F (49) | CHD, UAP, Killip I, arrhythmia | 0.5 | 4.06 | 1.97 | 2.04 | Atorvastatin, aspirin, Yiqi Fumai | MNC^a^  Serum^b^ |
| **4** | M (85) | CHD, ACS, ICM, Killip III, hypertension (III), hyperlipidemia | 1.59 | 5.63 | 3.59 | 1.31 | Atorvastatin, nifedipine, aspirin, furosemide, spirolactone, safflower yellow, benazapril | MNC^a^  Serum^b^ |
| **5** | M (62) | CHD, AMI, Killip I, hyperlipidemia | 1.92 | 5.52 | 3.49 | 1.27 | Rosuvastatin, aspirin, plavix, clexane, potassium magnesium aspartate | MNC^a^  Serum^b^ |
| **6** | F (78) | CHD, Killip III, arrhythmia, hypertension (III), AHCM, CI, CAS | 0.81 | 3.74 | 2.25 | 1.17 | Atorvastatin, aspirin, levamlodipine, metoprolol, Yiqi Fumai, isoket, clexane | MNC^a^  Serum^b^ |
| **7** | F (72) | CHD, AMI, Killip I, hypertension (III), hyperlipidemia, DM | 2.09 | 6.86 | 4.26 | 1.36 | Atorvastatin, clexane, aspirin, plavix, benazapril, metoprolol, furosemide, spirolactone, potassium magnesium aspartate, amiodarone, acetylcysteine | MNC^a^  Serum^b^ |
| **8** | M (82) | CHD, AMI, Killip II, hypertension (II), HHD, AECO-PD, respiratory failure (type II), KC, MA | 0.68 | 3.17 | 1.55 | 1.41 | Atorvastatin, aspirin, plavix, isoket, SM, acetylcysteine, doxofylline, ambroxol , BDP, methylprednisolone, kedacillin | MNC^a^  Serum^b^ |
| **9** | M (77) | CI, hypertension (III) | 0.63 | 4.11 | 2.45 | 1.12 | Atorvastatin, aspirin, plavix, clexane, gingko, citicoline, NBP, levamlodipine, edaravone, Wuling capsule, GDL, CBC | MNC^a^  Serum^b^ |
| **10** | M (64) | CI, hypertension (II), CS, MA, hepatic injury | 1.27 | 3.46 | 1.99 | 1.02 | Aspirin, plavix, gingko, citicoline, edaravone, CBC, GSH | MNC^a^  Serum^b^ |
| **11** | M (59) | CI, CS, B12 and folate deficiency, hyperhomocysteinemia, hypoalbuminemia | 0.74 | 4.54 | 2.74 | 1.2 | Atorvastatin, gingko, citicoline, aspirin, plavix, aceglutamide, mecobalamin, vitamin B1, alprostadil, ceftezole sodium, acetylcysteine | MNC^a^  Serum^b^ |
| **12** | M (53) | HAII, hyperlipidemia | 2.22 | 6.10 | 3.94 | 1.20 | Atorvastatin, gingko, clexane, edaravone, silybinin | MNC^a^  Serum^b^ |
| **13** | F (83) | CHD, AMI, Killip II, arrhythmia, hyperlipidemia, DM | 1.19 | 5.08 | 3.06 | 1.25 | Rosuvastatin, ozagrel, panax ginseng, MAAPN, aspirin, norvasc, atacand, NBP, probucol | Serum^b^ |
| **14** | F (53) | CHD, AMI, Killip I, DM, hyperlipidemia | 1.25 | 5.42 | 3.37 | 1.34 | Atorvastatin, aspirin, plavix, SM, clexane, isoket, metoprolol | Serum^b^ |
| **15** | F (74) | CHD, AMI, Killip I, DM, hyperlipidemia | 1.46 | 5.13 | 3.32 | 1.11 | Atorvastatin, aspirin, plavix, potassium magnesium aspartate, clexane, furosemide, spirolactone, metoprolol | Serum^b^ |
| **16** | F (70) | CI, CAS, DM, hyperlipidemia | 2.16 | 5.71 | 3.31 | 1.51 | Atorvastatin, aspirin, plavix, gingko, citicoline, NBP | Serum^b^ |
| **17** | M (81) | CI, CAS, hypertension (II), hyperlipidemia | 1.13 | 5.43 | 3.25 | 1.23 | Atorvastatin, aspirin, NBP, edaravone, vinpocetine | Serum^b^ |
| **18** | M (85) | CHD, AMI, Killip I, hypertension (III), DM (type II), hyperlipidemia | 2.61 | 4.58 | 2.82 | 0.82 | Atorvastatin, aspirin, plavix, potassium magnesium aspartate, clexane, benazapril, metoprolol | Serum^b^ |
| **19** | F (82) | CI, arrhythmia, hyperlipidemia | 2.62 | 4.13 | 2.00 | 0.92 | Aspirin, plavix, NBP, citicoline, gingko, mecobalamin | Serum^b^ |
| **20** | M (68) | CI, CAS, hypertension (III), DM, HHcy, hyperlipidemia | 2.50 | 4.56 | 2.52 | 0.99 | Atorvastatin, aspirin, plavix, gingko, NBP, citicoline, mecobalamin, metformin | Serum^b^ |
| **21** | M (59) | CI, CAS, hypertension (III) | 1.61 | 3.82 | 2.49 | 0.91 | Atorvastatin, aspirin, plavix, gingko, citicoline, alprostadil, Piracetam, SM, NBP | Serum^b^ |
| **22** | F (69) | CHD, AMI, Killip I, hyperlipidemia | 3.90 | 7.37 | 4.76 | 1.04 | Atorvastatin, aspirin, spirolactone, papaverine, entresto, betaxolol, clopidogrel, lovenox | MNC^c^  Plasma^d^ |
| **23** | M (62) | AMI, Killip I, hyperlipidemia | 1.20 | 5.34 | 3.28 | 1.14 | Rosuvastatin, aspirin, papaverine, clopidogrel, lovenox, niacin, irbesartan | MNC^c^  Plasma^d^ |
| **24** | M (37) | AMI, Killip I, hyperlipidemia | 6.88 | 5.57 | 3.14 | 0.76 | Atorvastatin, aspirin, clopidogrel, clexane, bivalirudin, irbesartan, betaxolol, cilostazol | MNC^c^  Plasma^d^ |
| **25** | M (61) | AMI, hypertension (II), DM (type II), hyperlipidemia | 2.27 | 8.01 | 5.30 | 1.13 | Atorvastatin, aspirin, clexane, spirolactone, papaverine, furosemide, entresto, clopidogrel, dapagliflozin, ezetimibe, | MNC^c^  Plasma^d^ |
| **26** | M (66) | UAP, Killip II, DM, hyperlipidemia | 4.55 | 6.40 | 3.78 | 1.27 | Atorvastatin, aspirin, papaverine, ticagrelor, betaxolol, irbesartan, norvasc | MNC^c^  Plasma^d^ |
| **27** | F (71) | AMI, hyperlipidemia | 1.81 | 5.53 | 3.58 | 0.89 | Atorvastatin, aspirin, clopidogrel, papaverine, spirolactone, furosemide, potassium magnesium aspartate | MNC^c^  Plasma^d^ |
| **28** | M (60) | Healthy donor | 1.67 | 4.94 | 2.90 | 1.84 | None | Plasma^d^ |
| **29** | F (51) | Healthy donor | 1.07 | 3.71 | 2.33 | 1.04 | None | Plasma^d^ |
| **30** | F (41) | Healthy donor | 0.43 | 2.68 | 2.10 | 1.38 | None | Plasma^d^ |
| **31** | M (39) | Healthy donor | 0.85 | 2.79 | 2.86 | 1.72 | None | Plasma^d^ |

F, female; M, male; MNC, mononuclear cells; TG, triglyceride; TC, total cholesterol; LDL-c, low-density lipoprotein cholesterol; HDL-c, high-density lipoprotein cholesterol; CHD, coronary heart disease; AMI, acute myocardial infarction; ACS; acute coronary syndrome; DM, diabetes mellitus; AU, aortic ulcer; IMH, aortic intramural hematoma; ICM, ischemic cardiomyopathy; UAP; unstable angina; AHCM, apical hypertrophic cardiomyopathy; AECO-PD; acute exacerbation of chronic obstructive pulmonary disease; HHD, hypertensive heart disease; KC, kidney calculus; MA, metabolic acidosis; CRF, chronic renal failure; CI, cerebral infarction; CAS, cerebral arterial stenosis; HAII, hemorrhage after an acute ischemic stroke; CS, cerebrovascular stenosis; HHcy, hyperhomocysteinemia; MAAPN , muscular amino acids and peptides and nucleosides; NBP, Dl-3-n-butylphthalide; SM, salvia miltiorrhiza; GDL, ginkgo diterpene lactones; BDP; beclomethasone dipropionate; CBC, coenzyme B complex; GSH, reduced glutathione

^a^Peripheral blood (PB) mononuclear cells for ex vivo experiments (oxLDL treatment).

^b^PB serum for cytometric bead array (CBA) of cytokines.

^c^PB mononuclear cells for ex vivo experiments (nLDL treatment).

^d^PB plasma for ELISA-based analysis of oxLDL.

**Supplemental figure legends**

**Figure S1. Pro-inflammatory (M1-like) macrophages are more predominant than anti-inflammatory (M2-like) macrophages in AS lesions.** (**a**, **b**) Frozen sections of the embolus samples obtained from additional two patients with IS via embolectomy were stained with hematoxylin and eosin (HE) or immunohistochemically (IHC) stained for CD68 (macrophage/M1 marker) and CD163 (M2 marker). Representative images for two patient samples were shown (left; scale bar = 200 μm). Areas outlined by dashed line indicate blood clots. For the IHC images, integrated option density (IOD) was measured on three fields per sample using Image J software. Average optical density (AOD) was then calculated using the formula AOD = IOD / area (right; two-tailed Student's t-test).

**Figure S2. OxLDL exposure leads to transcriptional reprogramming of macrophages.** The R2: Genomics Analysis and Visualization Platform (GEO ID: GSE54666) was used to survey the genome-wide gene expression profile (GEP) in primary human monocyte-differentiated macrophages (by 50 ng/ml M-CSF) after exposure to 50 μg/ml oxLDL for 48 hrs. (**a**) Heatmap illustrates a unique GEP (log2 fold change; cutoff, *P* < 0.05) of macrophages after exposure to oxLDL vs. vehicle (Veh; n = 6 for each condition). (**b**, **c**) KEGG PathwayFinder analysis (one-way ANOVA, cutoff, *P* < 0.05) was performed to demonstrate alterations of the PPAR pathway (**b**) and steroid biosynthesis (**c**). (**d-f**) Expression (log2) of the representative genes for M1 (**d**), M2 and its subtypes (M2a-d; **e**), as well as other phenotypes of macrophages (**f**), including those related to AS [e.g., Mox (HMOX1 encoding HO-1, SRXN1, and TXNRD1) and Mhem (NR1H3 encoding LXRα)] or observed in AS lesions by scRNAseq [e.g., TREM2^hi^ foamy macrophages (e.g., TREM2, CTSD, LGALS3, and FABP4), inflammatory macrophages (e.g., NFKBIZ, NLRP3, CEBPB, and CXCL2), and resident-like macrophages (e.g., F13A1, TXNIP, SEPP1, and PF4)] was compared between Veh and oxLDL treatment using (log2, one-way ANOVA).

**Figure S3. Exposure to oxLDL resulted in phenotypic and metabolic changes in macrophages.** (**a**) Peripheral blood monocytes from patients with CHD or IS were differentiated to macrophages by 50 ng/ml M-CSF, after which the percentage of CD14^+^ macrophages were determined using flow cytometry, indicating high purity of macrophages (> 90%). (**b**) Macrophages differentiated from primary monocytes of additional two patients with CHD or IS were incubated with 5 or 10 μg/ml oxLDL for 24 hrs, after which images were captured under inverted microscopy (scale bar = 20 μm). (**c**) Peripheral blood monocytes from patients with CHD were differentiated to macrophages by 50 ng/ml M-CSF, followed by incubation with 10 μg/ml nLDL for 24 hrs. After treatment, qPCR analysis was performed to monitor the expression of IL-1β, TNF-α, MCP-1, VEGF, and CD206. (**d**, **e**) Using the R2: Genomics Analysis and Visualization Platform (GEO ID: GSE54666), heatmap illustrated DEGs (one-way ANOVA, cutoff, *P* < 0.05 ) enriched for OXPHOS (upper), TCA cycle (lower left), and glycolysis/gluconeogenesis (lower right) after exposure to 50 μg/ml oxLDL vs. vehicle (Veh) for 48 hrs (n = 6 for each condition) in primary human monocyte-differentiated macrophages (**d**). Dashed line square indicates the genes that were relatively down-regulated (for OXPHOS and TCA cycle) or up-regulated (for glycolysis/gluconeogenesis) in response to oxLDL. The representative genes for OXPHOS (e.g., *NDUFA6* and *ATP6V1E1*) and TCA (e.g., *IDH1* and *IDH2*) or glycolysis (e.g., *HK1*, *HK2*, *ALDH3A2*, and *ALDH9A1*) were compared between Veh and oxLDL treatment (**e**; log2, one-way ANOVA).

**Figure S4. OxLDL activates both NF-κB and HIF pathways in macrophages.** (**a**) The R2: Genomics Analysis and Visualization Platform [GEO ID: GSE54972] was used to analyze the enrichment of H3K27Ac at the loci of *ARNT*, *HIF1A*, *EPAS1*, *CHUK*, and *RELA* by ChIP-seq in primary human monocyte-differentiated macrophages (by 50 ng/ml M-CSF) exposed to 50 μg/ml oxLDL for 48 hrs. TSS, transcriptional start site. (**b**) RAW264.7 cells exposed to 25-100 μg/ml oxLDL for 2 hrs or 50 μg/ml oxLDL for 0.5-2 hrs (see Figure 4c). Blots of total and phosphorylated p65 were quantified using Image J software. Quantification data from three independent experiments were compared using two-tailed Student's t-test. (**c**) THP-1-derived macrophages (differentiated by 50 ng/ml PMA for 48 hrs, followed by incubation without PMA for 24 hrs) were exposed to 50 μg/ml oxLDL for 0.5 - 2 hrs. After treatment, Western blot analysis was performed to monitor phosphorylation of p65 and expression of HIF-1α and HIF-1β. (**d**) Raw264.7 cells were exposed to 25 - 100 μg/ml nLDL for 24 hrs (left) or 50 μg/ml nLDL for 0.5 - 2 hrs (right), after which Western blot analysis was performed to monitor the activation of NF-κB and HIF pathways. (**e**) RAW264.7 cells and THP-1-derived macrophages exposed to 50 μg/ml nLDL or oxLDL for 24 hrs (see Figure 4e). Blots of total and phosphorylated p65 were quantified and analyzed as described above.

**Figure S5. NF-κB inhibition prevents oxLDL-induced HIF activation and morphological/functional alterations.** (**a**) Raw264.7 cells were pre-treated with 5 μM IKK2 inhibitor IV for 2 hrs, followed by exposure to 50 μg/ml oxLDL for additional 24 hrs, after which Western blot analysis was performed to monitor the activation of NF-κB and HIF pathways. β-actin was probed as loading control. (**b**) Peripheral blood monocytes from additional three patients with CHD or IS were differentiated to macrophages by 50 ng/ml M-CSF, followed by incubation with 10 μg/ml oxLDL for 24 hrs after pre-treatment with 10 μM PTL for 2 hrs. After treatment, images were captured under inverted microscopy (scale bar = 20 μm). (**c**) KEGG analysis of *ARNT*-related DEGs enriched for OXPHOS and TCA in primary human monocyte-differentiated macrophages after exposure to 50 μg/ml oxLDL vs. vehicle (Veh) for 48 hrs, using the R2: Genomics Analysis and Visualization Platform (GEO ID: GSE54666). Red square indicates DEGs negatively correlated with *ARNT*. (**d**) THP-1 cells were incubated with or without 10 μM PTL for 2 hrs, followed by exposure to 50 μg/ml oxLDL for 24 hrs, after which cells were washed twice and incubated for 3 days. Cells were then re-stimulated with low dose of LPS (10 ng/ml) for 24 hrs, after which qPCR was performed to determine the expression of IL-1β and TNF-α. Values represent the means ± SD for at least three independent experiments (two-tailed Student's t-test). For each experiment, the readout of vehicle treatment as control is arbitrarily set to 1.0.

**Figure S1**

**Figure S2**

**Figure S3**

**Figure S4**

**Figure S5**
